# Coevolution and Functional Effects of Endosymbiotic *Rickettsia* in *Leptocybe invasa* Fisher & LaSalle (Hymenoptera: Eulophidae) Across China

**DOI:** 10.1002/ece3.73066

**Published:** 2026-02-10

**Authors:** Xiu Xu, Leming Zhou, Jinting Xie, Junjue Li, Chunhui Guo, Zhende Yang

**Affiliations:** ^1^ Guangxi Colleges and Universities Key Laboratory for Cultivation and Utilization of Subtropical Forest Plantation, Guangxi Key Laboratory of Forest Ecology and Conservation, College of Forestry Guangxi University Nanning China; ^2^ Ecological Environment Monitoring and Scientific Research Center, Yellow River Basin Ecology and Environment Administration Ministry of Ecology and Environment Zhengzhou China

**Keywords:** antibiotic, coevolution, functional analysis, *L*. *invasa*, *rickettsia*

## Abstract

*Rickettsia* is an endosymbiotic bacterium that infects various arthropods, affecting the host's biology, ecology, and evolution. *Leptocybe invasa* is an invasive pest that severely damages eucalyptus plants. A comprehensive investigation of *Rickettsia* in 313 female *L. invasa* individuals from 17 Chinese populations revealed a 100% infection prevalence. Sequencing of three host molecular markers—mitochondrial *COI*, nuclear *ITS*, and *28S*—led to the identification of a novel *L. invasa* haplotype, designated Haplotype 1 × 2, which exhibits mito‐nuclear discordance. Concurrently, sequencing of four *Rickettsia* genes (*16S* rRNA, *gltA*, *atpA*, *rpmE*) revealed two distinct strains, termed STRiA and STRiB. These strains demonstrated a specific association with the host lineages, where STRiA was exclusively associated with lineage A (comprising Haplotype 1 and Haplotype 1 × 2), and STRiB was linked to lineage B. Phylogenetic analysis of the multigene datasets from both the host and *Rickettsia* revealed a high degree of topological congruence between their inferred trees. Correlation analysis further demonstrated a moderate positive association (*r* = 0.307). The significance of this relationship was supported by a Mantel test (*p* < 0.005), suggesting coevolution. Low‐dose tetracycline treatment effectively eliminated *Rickettsia* from *L. invasa*. *L. invasa* treated with tetracycline exhibited a significantly higher proportion of male offspring, reduced *Rickettsia* expression, and decreased body length and lifespan in female offspring. Transcriptome analysis comparing *Rickettsia*‐free and *Rickettsia*‐infected *L. invasa* following antibiotic treatment identified 178 differentially expressed genes (122 up‐regulated, 56 down‐regulated). These genes were enriched in GO terms related to metabolic processes, cellular processes, cellular components, binding functions, and catalytic activities. KEGG pathway analysis revealed enrichment of differentially expressed genes primarily in metabolic pathways, insect hormone biosynthesis, and thermogenesis. Additionally, enrichment was observed in key signaling pathways, including Ras, MAPK, NF‐κB, TGF‐β, TNF, and Apelin. These findings elucidate the coevolutionary relationship and functional roles of *Rickettsia* in *L. invasa*, providing a foundation for symbiont‐mediated biological control.

## Graphical abstract

Two distinct lineages of *L. invasa* and the dominant endosymbiont, *Rickettsia*, was identified, followed by an analysis of its infection density across various developmental stages and tissues of *L. invasa*. Furthermore, two distinct strains of the *Rickettsia* endosymbiont were detected, and the phylogenetic relationships between these strains and their *L. invasa* hosts were characterized. *L. invasa* was treated with tetracycline. The effects of *Rickettsia* infection on the reproduction, offspring sex ratio, and lifespan of *L. invasa* were investigated. Finally, transcriptome sequencing was performed to reveal genetic changes in *L. invasa* following antibiotic‐mediated removal of *Rickettsia* and to explore the functional mechanisms of this endosym‐biont within its host.

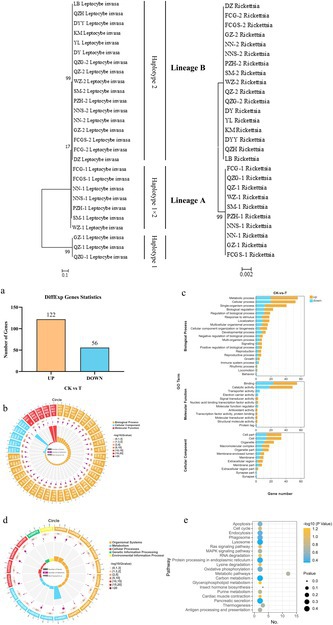

## 1 Introduction

Biologically invasive species represent a major global environmental problem threatening biodiversity, causing significant ecological, economic, and social losses worldwide (Pyšek et al. 2020). Symbiotic relationships represent a pervasive phenomenon among arthropods, with symbioses between invasive insects and bacteria serving as key drivers of invasion success (Liu et al. 2022). The concept of “symbiotic invasion” in insect‐endosymbiont systems has recently emerged as a significant research focus in pest invasion mechanisms (Lu et al. 2016). Symbiotic bacteria in invasive insects have long been implicated in host adaptation and dispersal, and numerous studies have demonstrated the importance of insect‐endosymbiont interactions (Frago et al. 2012). For instance, phenols produced by *Pantoea agglomerans* , an endosymbiont of *Schistocerca gregaria*, exhibit antibacterial properties and reduce pathogenic fungal invasion, whereas locusts lacking this symbiont show heightened susceptibility to pathogenic fungal infections (Engel and Moran 2013). Volatile compounds from symbiotic bacteria in *Dendroctonus valens* mitigate antagonistic interactions by modulating carbon source utilization hierarchies, facilitating beetle invasion and colonization (Xu et al. 2016; Zhou et al. 2017).

Endosymbiotic bacteria and their insect hosts establish distinct symbiotic relationships, including obligate and facultative modes, which have been investigated to elucidate their symbiotic history and co‐evolutionary relationships under diverse conditions (Nakabachi and Suzaki 2023; Valerio et al. 2024). Most arthropods harbor maternally inherited endosymbionts (vertical transmission), establishing a correlation between mitochondrial genomes and female‐heterogametic systems (Hurst and Jiggins 2005). This association can drive the spread of mtDNA variants, resulting in skewed frequency distributions of mtDNA alleles (Atyame et al. 2011; Schuler et al. 2016). Consequently, endosymbionts play essential roles in host mtDNA evolution and phylogeny (Bennett and Moran 2015; Schuler et al. 2018). In the peach aphid, *Myzus persicae* , the phylogeny of *Buchnera aphidicola* —inferred from *trpB*, *dnaN*, and *trpEG* sequences—was found to be largely congruent with that of its host, supporting their coevolutionary relationship (Wu et al. 2008). Similarly, in *Brontispa longissima* , the phylogenies of *Wolbachia* (on the basis of wsp and multilocus sequence typing) and host mtDNA showed strong topological concordance, also indicating coevolution. Additionally, *Wolbachia* infection was shown to influence the genetic structure and diversity of the host population (Ali et al. 2018). Thus, phylogenetic analysis of invasive pests and their endosymbionts has important implications for adaptive evolution and ecology, and is crucial for developing effective pest management strategies (Prasannakumar et al. 2025; Xu et al. 2023).

*Leptocybe invasa* Fisher & LaSalle (Hymenoptera: Eulophidae: Tetrastichinae) is a devastating invasive pest that damages Eucalyptus plantations (Mendel et al. 2004; Ngoc Hoan Le et al. 2018; Zheng, Li, et al. 2014). Adults damage tender plant tissues through feeding and oviposition, inducing galls that ultimately cause tree mortality. Originating in Australia, *L. invasa* was first documented in the Middle East and Mediterranean region in 2000 and has since spread to 45 countries across Asia, Europe, Africa, Oceania, and America (Mendel et al. 2004; Ngoc Hoan Le et al. 2018; Zheng, Li, et al. 2014). Because of intensifying global warming and increased human activities, even traditionally unsuitable Chinese regions are projected to become susceptible to *L. invasa* invasion (Huang et al. 2019). In China, this species was first detected in 2007 in Dongxing City, Guangxi Zhuang Autonomous Region. Subsequently, it spread rapidly throughout southern, southeastern, and southwestern China, including Guangxi, Guangdong, Hainan, Taiwan, Fujian, Jiangxi, Hunan, Sichuan, and Yunnan provinces—regions characterized by warm, humid climates supporting extensive Eucalyptus cultivation (Huang et al. 2019; Mendel et al. 2004; Zheng, Yang, et al. 2014). Geographically distinct *L. invasa* populations exhibit genetic differentiation on the basis of *COI* sequences and microsatellite markers, with two lineages identified. Lineage A comprises populations from North America, Africa, and Europe, whereas lineage B dominates in Australia, China, and Southeast Asia (Dittrich‐Schröder et al. 2018; Gevers et al. 2021; Guo et al. 2020; Nugnes et al. 2015).

*Rickettsia*, a primary symbiotic bacterium primarily localized within female *L. invasa* ovaries, is maternally transmitted to offspring (Guo et al. 2020; Nugnes et al. 2015). This bacterium significantly influences host reproductive regulation, inducing phenotypes such as parthenogenesis and male killing (Giorgini et al. 2010; Liu et al. 2024; Majerus and Majerus 2010). As observed in other systems, infection with *Rickettsia* in *Neochrysocharis formosa* and *Pnigalio soemius* results exclusively in female offspring (Giorgini et al. 2010). Conversely, elimination of the bacterium through antibiotic treatment enables these wasps to produce substantial numbers of male offspring. Currently, two closely related *Rickettsia* strains have been identified, each infecting a putative cryptic species of *L. invasa* and associated with distinct sex ratios (Nugnes et al. 2015). *Rickettsia* has also been implicated in influencing host mtDNA evolution. Thongprem et al. reported identical mtDNA haplotypes shared between two distinct species, *Coleonema puella* and *C. pulchellum* , a pattern hypothesized to result from *Rickettsia*‐mediated interspecific transfer of mtDNA during a rare hybridization event. This suggests that, much like *Wolbachia*, *Rickettsia* may act as a genetic vector capable of facilitating mtDNA introgression and shaping its evolutionary trajectory (Thongprem et al. 2021). From a theoretical perspective, Gregory posited that infection of a host population by one or more endosymbionts can alter mitochondrial polymorphism patterns through selection acting on the symbionts. Depending on the recency of invasion and symbiont prevalence, such infections may either reduce or increase mitochondrial diversity, and can also shift the frequency distribution of haplotypes within the population (Hurst and Jiggins 2005). Therefore, *Rickettsia*'s influence on wasp mtDNA variation cannot be ignored, and nuclear gene data are essential for verifying mtDNA‐based findings (Adachi et al. 2008; Tseng et al. 2019), enabling enhanced understanding of invasion mechanisms and improved prevention, management, and control strategies.

The functional analysis of intracellular bacteria presents significant challenges, primarily because of their obligate dependence on the host. To investigate these regulatory mechanisms, antibiotic treatment is commonly employed to generate asymbiotic or low‐titer host models. For instance, Kopal et al. (Singhal and Mohanty 2025) successfully eliminated *Wolbachia* from *Drosophila melanogaster* via tetracycline treatment, which resulted in impaired host survival and reproductive capacity. Alternative non‐antibiotic methods, including high‐temperature exposure, intensive rearing, and nutritional stress, can also achieve a reduction in bacterial titer (Ross et al. 2016). However, these approaches often fail to completely eradicate bacterial cells and are prone to reinfection upon the restoration of normal conditions (Gunderson et al. 2020). Consequently, antibiotic treatment remains the most widely utilized method for clearing symbionts (Wang et al. 2020). Following the generation of such models, transcriptome analysis has proven effective in revealing how endosymbionts reshape host gene expression. For example, Hu et al. (2024) identified 843 differentially expressed genes in *Wolbachia*‐infected female whiteflies. The KEGG pathways enriched for these genes included those involved in detoxification, redox and metabolic processes, as well as immune signaling pathways such as those for cell adhesion molecules, phagosomes, endocytosis, lysosomes, MAPK, Toll/Imd, and mTOR. Similarly, Li et al. (2024) showed that *Rickettsia* regulates key metabolic and nutrient synthesis pathways in its whitefly host. Despite these advances, the functional mechanisms of *Rickettsia* in *L. invasa*—particularly how it molecularly modulates host reproduction, development, and invasion adaptation—remain poorly understood.

To address this knowledge gap, the present study integrates phylogenetic, experimental, and transcriptomic approaches. We first investigated *Rickettsia* infection prevalence and strain diversity across 17 Chinese populations of *L. invasa* and evaluated the coevolutionary relationship between host and symbiont using mitochondrial, nuclear, and multiple *Rickettsia* gene markers. We then applied low‐dose tetracycline to eliminate *Rickettsia* and assessed its effects on host reproduction, offspring sex ratio, and longevity. Finally, transcriptome sequencing of *Rickettsia*‐free and *Rickettsia*‐infected wasps was performed to identify differentially expressed genes and enriched pathways, thereby elucidating the molecular mechanisms underlying *Rickettsia*‐mediated regulation in *L. invasa*. This multi‐level approach aims to clarify not only the evolutionary association between *L. invasa* and *Rickettsia* but also the molecular functional basis of this symbiosis in promoting host invasion success, offering insights for future symbiont‐based management strategies.

## 2 Materials and Methods

### 2.1 Insect Sample Collection

**FIGURE 1:**
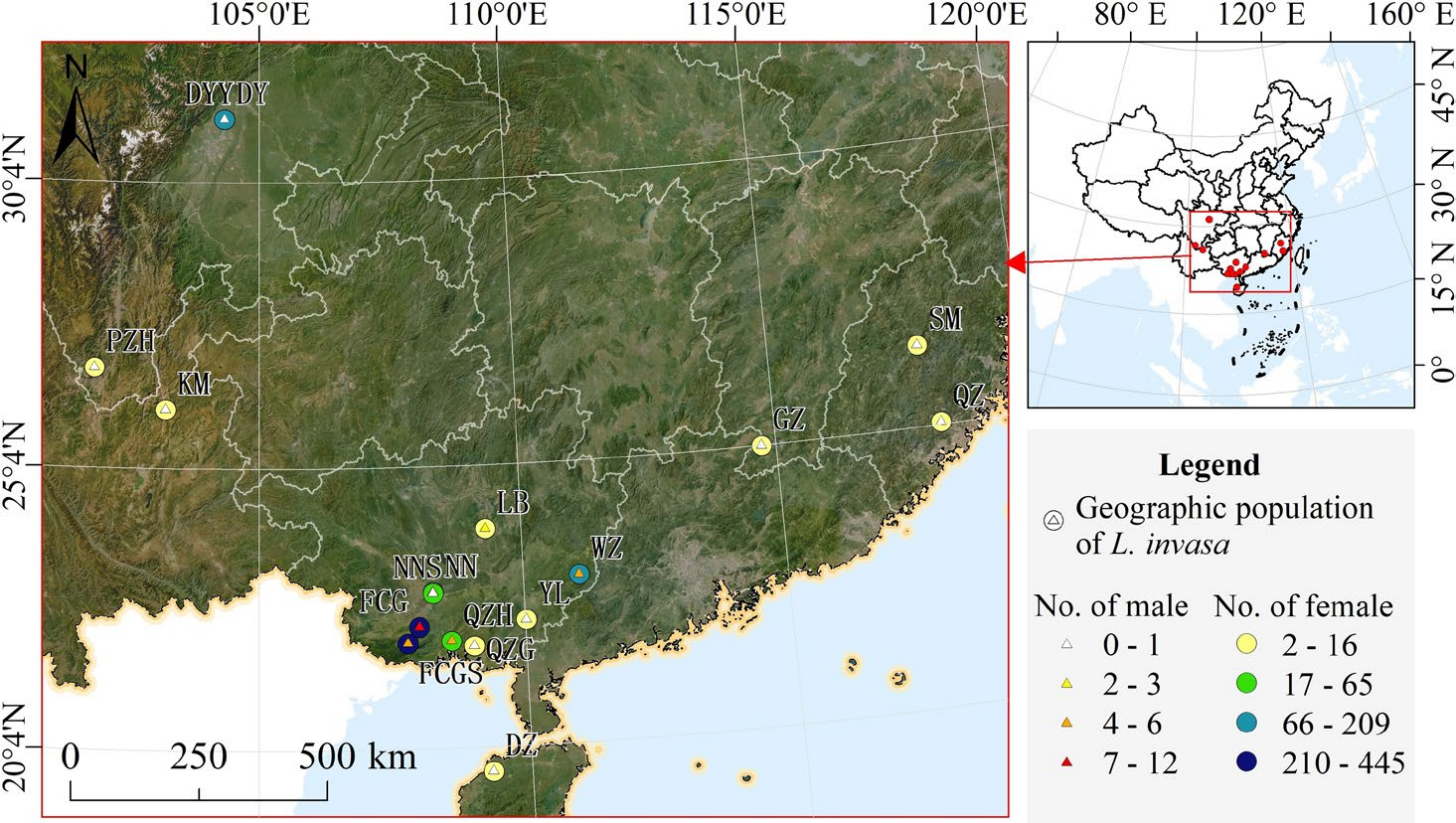
Geographic distribution of the 17 populations of *L. invasa*.

Gall‐infested branches were collected from Eucalyptus plantations across diverse geographical regions in China (Figure 1, Table 1, Appendix S1), transported to the laboratory in water‐filled plastic bottles, maintained, and housed within sealed net cages (40 cm × 40 cm × 60 cm) to prevent adult *L. invasa* escape. All materials were maintained under controlled laboratory conditions: a temperature of 27°C ± 1°C (reflecting average temperature during collection), a 16:8 h light: dark cycle, and a relative humidity of 70%–80%. To preserve branch freshness, water in the glass bottles was replaced daily until eucalyptus gall wasp emergence ceased. Following emergence, adult wasps were collected daily and preserved in 10 mL centrifuge tubes at −20°C. Newly emerged wasps were subsequently preserved in 95% ethanol and morphologically identified for sex determination following Zheng, Li, et al. (2014).

**TABLE 1:** Geographic population of *L. invasa*.

Population	Location	Longitude (E)	Latitude (N)	Time	No. of female	No. of male	Host
DY	Deyang, Sichuan	104.28	31.17	2019/12/4	38	0	*E. camaldulensis*
FCG	Fangchenggang, Guangxi	108	22.23	2019/7/18	445	12	*E. grandis × E. tereticornis*
FCGS	Fangchenggang, Guangxi	107.78	21.95	2019/10/12	312	6	*E. grandis × E. tereticornis*
GZ	Ganzhou, Jiangxi	114.72	25.06	2019/9/8	4	0	*E. robusta*
NN	Nanning, Guangxi	108.28	22.85	From 2018–6 to 2018–8	65	5	*E. grandis × E. tereticornis*
NNS	Nanning, Guangxi	108.28	22.83	From 2019–6 to 2019–8	33	1	*E. exseria*
PZH	Panzhihua, Sichuan	101.75	26.82	2018/7/27	12	0	*E. camaldulensis*
QZ	Quanzhou, Fujian	118.22	25.11	2018/8/13	2	0	*E. grandis × E. tereticornis*
SM	Sanming, Fujian	117.95	26.5	2019/9/11	8	0	*E. grandis × E. tereticornis*
WZ	Wuzhou, Guangxi	111.05	23.05	2016/7/31	161	5	*E. grandis × E. tereticornis*
DZ	Danzhou, Hainan	109.28	19.62	2016/11/28	4	0	*E. exseria*
QZG	Qinzhou, Guangxi	109.02	21.87	2019/7/6	6	0	*E. grandis × E. tereticornis*
YL	Yulin, Guangxi	110.01	22.3	2019/8/12	3	1	*E. grandis × E. tereticornis*
KM	Kunming, Yunnan	103.17	26.1	2019/7/30	3	0	*E. globulus*
DYY	Deyang, Sichuan	104.28	31.17	2019/12/5	209	1	*E. camaldulensis*
LB	Laibin, Guangxi	109.31	23.94	2020/7/7	16	3	*E. grandis × E. tereticornis*
QZH	Qinzhou, Guangxi	108.6	21.97	2020/6/30	32	5	*E. grandis × E. tereticornis*

*L. invasa* specimens for investigating the abundance of *Rickettsia* dynamics were collected from the teaching and experimental base of Guangxi University. Young larvae (20‐day‐old), mature larvae (40‐day‐old), pupae, and female adults aged 1‐day‐old, 3‐day‐old, 6‐day‐old, 9‐day‐old, and 12‐day‐old post‐emergence were selected and collected. One‐day‐old female adults were dissected under a stereomicroscope to separate head, thorax, and abdominal sections. Individual heads, thoraces, abdomens, and whole insects were each designated as one sample. Five biological replicates were prepared for each tissue type and developmental stage.

### 2.2 DNA Extraction

From each geographical population, 50 newly emerged (< 12 h) female *L. invasa* larvae were randomly selected. Following a 6‐h starvation period, specimens were subjected to sequential surface sterilization comprising three cycles of: (1) 60‐s immersion in sterile distilled water, and (2) 60‐s disinfection in 75% ethanol, with thorough drying between repetitions. Genomic DNA was extracted from individual specimens using a Chelex‐100 and proteinase K protocol (Guo et al. 2020). The concentration and purity of extracted DNA were detected by 1.2% agarose gel electrophoresis. Extracted DNA was stored at −20°C for subsequent analyses. Voucher specimens of *L. invasa* and corresponding DNA extracts were deposited in the Laboratory of Forest Conservation, College of Forestry, Guangxi University, Nanning, China.

### 2.3 Abundance of *Rickettsia* in Different Developmental Stages and Tissues of *L. invasa*

In this study, the *β‐actin* gene of *L. invasa* was used as an internal reference, and the *gltA* gene of *Rickettsia* was selected as the target. Specific primer pairs were designed for each gene (see Appendix S2 for sequences). The SYBR Green I method was used to determine the abundance of *Rickettsia* infection across various developmental stages and tissues of *L. invasa*. qRT‐PCR was conducted using the ChamQ Universal SYBR qPCR Master Mix (Vazyme Biotechnology Co. Ltd.), following the manufacturer's protocol, to quantify *Rickettsia* abundance. Amplifications were carried out on a LightCycler 480 system using a 20 μL reaction volume containing: 10 μL of 2× ChamQ Universal SYBR qPCR Master Mix, 0.4 μL each of the forward and reverse primers, 1 μL of template DNA, and ddH_2_O to volume. The amplification protocol consisted of an initial denaturation at 95°C for 30 s, followed by 40 cycles of denaturation at 95°C for 10 s and annealing/extension at 60°C for 30 s. Melting curve analysis was performed under the following conditions: denaturation at 95°C for 15 s, annealing at 60°C for 60 s, and a final extension at 95°C for 30 s. Following amplification, primer specificity was confirmed through analysis of the melting curve. The reliability of the internal reference gene and the designed primers was evaluated using the standard curve. The cycle threshold (*C*_t_) values for the *gltA* and *β‐actin* genes were recorded. The relative abundance of *Rickettsia* across different developmental stages and tissues of *L. invasa* was calculated using the $2^{-\Delta \Delta C_{t}}$ method (Daude et al. 2024). Statistical analyses were performed using the Least Significant Difference (LSD) method in SPSS software. For data with heterogeneous variances, the Games‐Howell post hoc test was applied to assess between‐group differences, with different lowercase letters denoting statistical significance.

### 2.4 PCR Amplification and Sequencing

The mitochondrial cytochrome oxidase I (*COI*) gene, the D2 expansion segment of the *28S* ribosomal RNA gene (*28S*‐D2), and the internal transcribed spacer 2 (*ITS2*) were amplified for *L. invasa*, along with the *Rickettsia 16S* rRNA gene, citrate synthase gene (*gltA*), ATP synthase F1 alpha subunit gene (*atpA*), and the *rpmE‐tRNAf* ^*Met*^ intergenic spacer. Three *L. invasa* genes and four *Rickettsia* loci were targeted for sequencing, with primer pairs listed in Appendix S2. PCR amplifications were performed in 25 μL reactions containing: 12.5 μL 2× Taq Plus PCR Mix (TianGen Biotech, Beijing), 1 μL DNA template, 9.5 μL deionized water, and 1 μL each of forward and reverse primers. Thermal cycling conditions comprised: initial denaturation at 94°C for 3 min; 30 cycles of denaturation at 94°C for 30 s, annealing at 53.0°C–59.5°C for 30 s, and extension at 72°C for 1 min; followed by final extension at 72°C for 5 min and indefinite hold at 4°C. PCR products were resolved on 1.0% agarose gels and visualized using a gel documentation system. Amplicons were bidirectionally sequenced by TsingKe Biological Technology (Beijing, China).

### 2.5 Molecular Phylogenetic Analysis

Sequencing chromatograms were visually inspected using ChromasPro v2.6.6 (https://technelysium.com.au/wp/chromaspro), and homology searches were performed using the NCBI BLAST (https://blast.ncbi.nlm.nih.gov/Blast.cgi) algorithm and EzBioCloud database (https://www.ezbiocloud.net), with reference sequences selected from diverse strains. Multiple sequence alignment was conducted using ClustalX v1.8.0 (https://www.clustal.org) with default parameters, followed by manual curation in BioEdit v7.0.7 (https://bioedit.software.informer.com) to trim low‐quality terminal regions. Consensus phylogenetic trees were reconstructed in MEGA v7.0 (https://www.megasoftware.net) using the Maximum Likelihood (ML) method, with tree credibility assessed through 1000 bootstrap replicates. Final tree visualization and annotation were performed using iTOL v6.0 (https://itol.embl.de). To assess the phylogenetic congruence between *L. invasa* and its endosymbiotic *Rickettsia*, a genetic distance matrix was first calculated using MEGA. The correlation between the host and endosymbiont genetic distance matrices was then determined using the CORREL function and analyzed with a Mantel test. The significance of the Mantel test correlation coefficient (*r*) was assessed using 200 permutation tests, with a significance threshold of *p* < 0.05. The *p*‐value was calculated as *P* = (*m* + 1)/(*n* + 1), where *m* is the number of permutations where *r* ≥ *r*‐obs (real *r*‐value), and *n* is the total number of permutations (200). Finally, a histogram of the *r*‐value distribution from the permutation test was generated using GraphPad Prism 9 software.

### 2.6 Antibiotic Feeding

Five commonly used antibiotics—rifampicin (RFP), ampicillin (AMP), tetracycline hydrochloride (TET), streptomycin sulfate (SM), and chloramphenicol (CAP)—were evaluated for efficacy and toxicity against *Rickettsia* in female *L. invasa*. All reagents, of analytical grade, were obtained from Nanning Guotuo Biotechnology. Antibiotics were dissolved in 10% honey water and tested at five concentrations: 1, 2, 3, 4, and 5 mg/mL.

Newly emerged (< 12 h) *L. invasa* females were transferred to 10 mL centrifuge tubes, with 10 specimens per tube. Six tubes were allocated per treatment (totaling 60 replicates). The control group received 10% honey water without antibiotics, whereas treatment groups received antibiotic‐supplemented 10% honey water. Conditions were maintained at 25°C ± 1°C, 75% ± 5% relative humidity, and a 16‐h light:8‐h dark photoperiod. Honey water was replenished daily. Thirty females per treatment were monitored for longevity assessment; the remaining 30 underwent DNA extraction for PCR‐based evaluation of *Rickettsia* elimination efficacy.

### 2.7 Eucalyptus Hydroponic Treatment

Tetracycline was dissolved in hydroponic solution, absorbed by eucalyptus seedling roots, and translocated systemically. This enabled *L. invasa* wasps to ingest the compound during feeding, thereby eliminating *Rickettsia*. The hydroponic nutrient solution was prepared according to Barros (de Barros et al. 2023). Treatment groups received tetracycline‐supplemented solution (final concentration: 1 mg/mL), whereas controls received antibiotic‐free solution. Nutrient solutions and dissolved oxygen were replenished every 2 days.

Experiments utilized 6‐month‐old *Eucalyptus grandis* × *E. urophylla* ‘DH201‐2’ tissue‐cultured seedlings (height: 25–30 cm; stem diameter: 2.5 mm) provided by Guangxi Academy of Forestry. Healthy, pest‐free seedlings were selected for uniformity. After acclimatization, each seedling was caged under 100‐mesh (60 × 40 cm) nets. Five newly emerged *L. invasa* females (collected from the teaching and experimental base of Guangxi University) were introduced per seedling for oviposition, with supplementary introductions of five females at 3‐day intervals (two additions total). Infection status of leaf veins, petioles, and branches was monitored every 48 h to confirm gall formation.

Thirty seedlings were assigned per treatment. After 10 days, seedlings were transferred to sealed mesh chambers (40 × 40 × 60 cm; 10 seedlings/chamber) under controlled conditions (25°C ± 2°C, 75% ± 5% RH, 16 L: 8D photoperiod). Upon gall maturation, newly emerged wasps were collected to quantify sex ratios, assess longevity, document morphology, and determine *Rickettsia* relative abundance. All quantitative data, including *Rickettsia* relative abundance (qPCR), offspring sex ratio (%), female body length, and longevity, were tested for normality (Shapiro–Wilk test) and homogeneity of variance (Levene's test) at *α* = 0.05. The sex ratio data, expressed as percentages, were subjected to arcsine square‐root transformation prior to parametric testing to meet normality and variance homogeneity assumptions. Transformed data were used in subsequent *t*‐tests. For datasets that violated variance homogeneity even after transformation, Welch's *t*‐test was applied. All statistical analyses were performed using SPSS 27.0 (IBM Corp., USA).

### 2.8 Total RNA Extraction and Transcriptome Sequencing

Newly emerged *L. invasa* females were collected from eucalyptus trees treated with 1 mg/mL tetracycline (treatment group, T) or antibiotic‐free medium (control group, CK), as described in Section 2.7. Each sample (200 individuals, *n* = 3 replicates/group) underwent total RNA extraction using Trizol Reagent (Invitrogen). RNA quality was verified through 1.2% agarose gel electrophoresis, Nanodrop 2000 spectrophotometry (A260/A280: 1.8–2.0; A260/A230: approximately 2.2), and Agilent Bioanalyzer 2100 assessment. Total RNA that met quality standards was used for sequencing library construction with the Illumina NEBNext Ultra RNA Library Prep Kit (NEB). The initial step involved the enrichment of mRNA using Oligo(dT) magnetic beads. The enriched mRNA was then fragmented, and first‐strand cDNA was synthesized using random hexamer primers and M‐MuLV Reverse Transcriptase. The RNA strand was subsequently degraded with RNase H, and the second cDNA strand was synthesized with DNA Polymerase I. The resulting double‐stranded cDNA was purified using 1.8X Agencourt AMPure XP Beads. The purified fragments underwent end repair, a poly(A)‐tailing reaction, and were then ligated to sequencing adapters. Library quality was assessed with the DNA 1000 Assay Kit (Agilent Technologies, 5067‐1504), and libraries passing quality control were sequenced in a paired‐end format on the Illumina HiSeq 2000 platform. Raw reads were processed using Trinity software to remove adapters, poly‐N sequences, and low‐quality bases, yielding clean data. Clean reads were assembled into unigenes, with Q20, Q30, and GC‐content metrics calculated. Subsequently, unigenes were functionally annotated against the Nr, Nt, Swiss‐Prot, KEGG, and COG databases. Gene expression levels were quantified using the FPKM method. Differentially expressed genes (DEGs) were screened using a significance threshold of |log_2_FC| ≥ 1 and FDR < 0.05, followed by KEGG pathway enrichment analysis to identify associated physiological and signaling pathways. To validate the transcriptome sequencing data, quantitative real‐time PCR was performed on total RNA from the sequenced samples. The *β‐actin* gene was used as an internal control. Reverse transcription was carried out using the PrimeScript RT Master Mix Kit (TaKaRa Bio Inc.) following the manufacturer's protocol. Reverse transcription of RNA into cDNA. Twelve DEGs were randomly selected, and their primers were designed using Primer 5.0 software; the sequences are provided in Appendix S5. Quantitative PCR was conducted using the TB Green Premix Ex Taq II (Tli RNaseH Plus) Kit (TaKaRa Bio Inc.) on a LightCycler 480 real‐time PCR system. For the qPCR reaction setup and thermal cycling protocol, refer to Section 2.3.

## 3 Results

### 3.1 Abundance of Rickettsia in *L. invasa* at Different Developmental Stages

**FIGURE 2:**
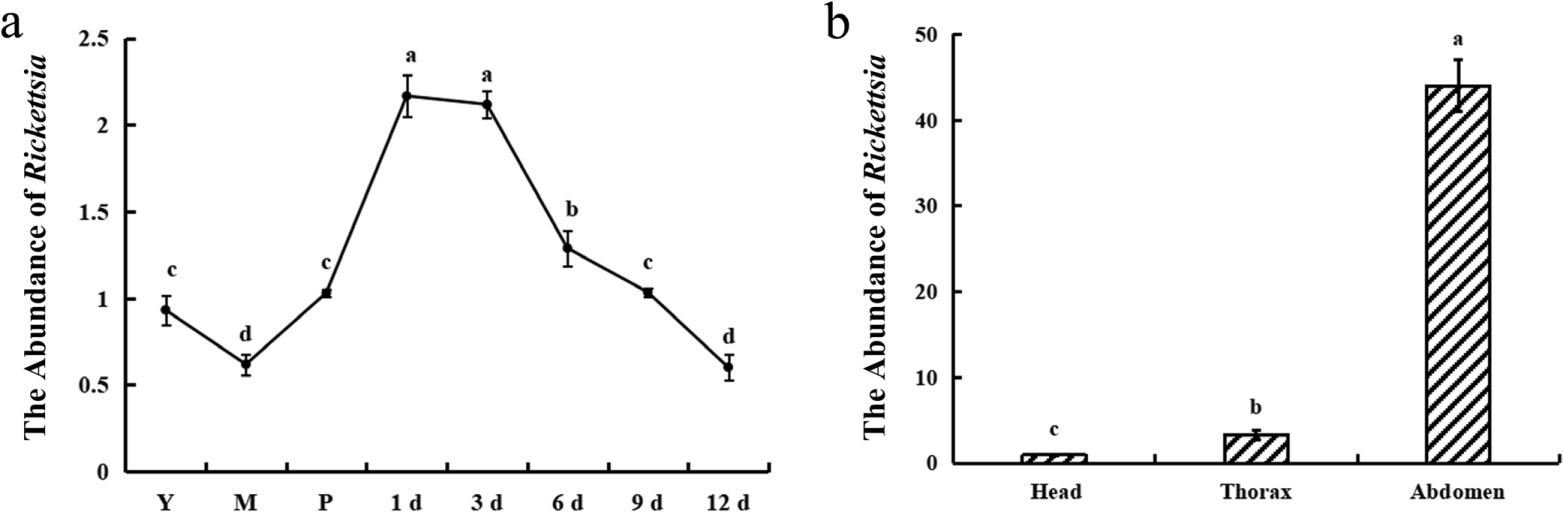
Copy number variation of *Rickettsia* infection abundance. (a) Relative abundance across different developmental stages of *L. invasa* (*n* = 5, *F* = 62.24, *p* < 0.05). Different lowercase letters denote statistically significant differences as determined by LSD test. (b) Relative abundance in different tissues of female *L. invasa* (*n* = 5, *F* = 185.85, *p* < 0.05). Because of heterogeneity of variance, differences were assessed using the Games‐Howell post hoc test, with different lowercase letters indicating statistical significance.

The *gltA* genes of the reference gene *β‐actin* and of the endosymbiont *Rickettsia* were analyzed as targets across specified developmental stages of *L. invasa* (lineage B): young larvae (20 days old), mature larvae (40 days old), pupae, and female adults at 1‐day‐old, 3‐day‐old, 6‐day‐old, 9‐day‐old, and 12‐day‐old post‐emergence. *Rickettsia* was detected in all life stages examined. One‐way ANOVA revealed a significant differential abundance of *Rickettsia* across developmental phases. Specifically, abundance in young larvae exceeded that in mature larvae, followed by progressive upregulation. Peak abundance was observed in 1‐day‐old and 3‐day‐old females, being significantly higher than at other stages, before declining steadily. By day 9, abundance was not significantly different from that in pupae. The lowest *Rickettsia* abundance was detected in 12‐day‐old females and mature larvae, with levels significantly lower than those in all other stages. (Figure 2a).

### 3.2 Abundance of Rickettsia in Different Tissues of *L. invasa*

Figure 2b presents the tissue‐specific distribution of *Rickettsia* copy numbers in *L. invasa* (lineage B). Abdominal tissues exhibited significantly higher *Rickettsia* abundance than both cephalic and thoracic tissues (*p* < 0.05), with thoracic abundance also substantially exceeding that in head tissues. These results indicate pronounced localization of *Rickettsia* within female abdominal tissues.

### 3.3 Lineage A of L. Invasa Was Founded in China

A total of 313 *COI* sequences (645 bp) were obtained, all of which were successfully translated without indels (Appendix S3). The sequences exhibited A/T bias with T + A content (65.35%) exceeding C + G content (34.65%), consistent with typical insect mitochondrial base composition (Table 2). A higher A + T content than G + C content was observed in all four *Rickettsia* genes, demonstrating an A + T compositional bias similar to that found in *L. invasa*.

**TABLE 2:** Basic information of gene sequenced of *Rickettsia* and *L. invasa*.

Gene	Host	C	V	Pi	S	Ts	Tv	V (%)	A + T (%)	G + C (%)	Fu's Fs	Tajima's *D*
*COI*	*L. invasa*	623	22	22	0	9	1	3.41	65.35	34.65	20.69	1.89
*28S*	*L. invasa*	555	1	1	0	0	0	0.18	42.98	57.02	−0.41	−0.41
*ITS2*	*L. invasa*	428	0	0	0	0	0	0	48.90	51.10	/	/
*16S rRNA*	*Rickettsia*	875	2	2	0	1	0	0.23	50.27	49.63	2.70	1.07
*gltA*	*Rickettsia*	498	1	1	0	0	0	0.20	66.53	33.47	/	/
*atpA*	*Rickettsia*	681	0	0	0	0	0	0	60.20	39.8	/	/
*rpmE‐tRNAf* ^*Met*^	*Rickettsia*	292	3	3	0	1	0	1.02	60.68	39.32	4.01	1.23
Concatenated	/	3952	29	29	0	11	1	0.73	/	/	/	/

Abbreviations: C, Conserved sites; Pi, Parsimony informative sites; S, Singleton variable sites; Ts, Transition sites; Tv, Transversion sites; V, Transition/Transversion bias; V, Variable sites.

**FIGURE 3:**
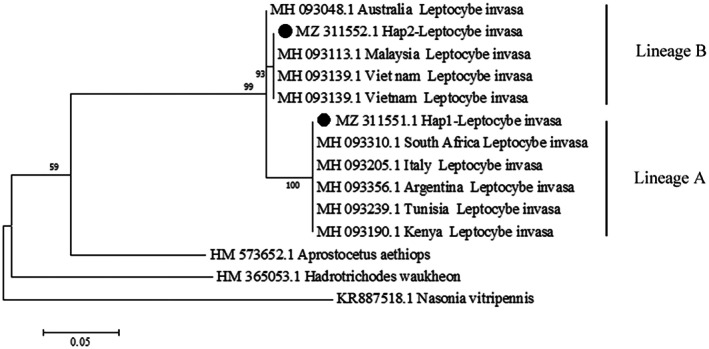
Phylogenetic tree of different *L. invasa* populations on the basis of *COI* sequences. This phylogenetic tree was constructed on the basis of an aligned sequence of 426 amino acid residues. Values shown at branch nodes represent bootstrap support on the basis of 1000 replicates.

Phylogenetic analysis identified two distinct lineages among *L. invasa* females in China (Figure 3) when compared with *Hadrotrichodes waukheon*, *Nasonia vitripennis*, and *Aprostocetus aethiops*. Lineage B (*n* = 238), representing 76% of specimens, was ubiquitous across all sampled populations and constitutes the dominant lineage in China. In contrast, Lineage A (*n* = 75), first reported in China, was restricted to NN, NNS, PZH, FCG, FCGS, QZ, GZ, SM, WZ, and QZG populations, comprising 24% of the total. Both lineages were also confirmed in male specimens (Table 3).

**TABLE 3:** Synthetic representation of the female *L. invasa* and its endosymbiont *Rickettsia* characterization.

Populations	*Leptocybe invasa*			*Rickettsia*					
	*COI* haplotype	*28S* haplotype	*ITS2* haplotype	Sequence type	*16S* haplotype	*gltA* haplotype	*atpA* haplotype	*rpmE‐tRNAf* ^*Met*^ haplotype	Number
FCG‐1	Hap1	H2	HI2	STRiA	D1	G1	A1	T1	30
FCG‐2	Hap2	H2	HI2	STRiB	D2	G2	A1	T2	2
FCGS‐1	Hap1	H2	HI2	STRiA	D1	G1	A1	T1	1
FCGS‐2	Hap2	H2	HI2	STRiB	D2	G2	A1	T2	31
GZ‐1	Hap1	H1	HI1	STRiA	D1	G1	A1	T1	1
GZ‐2	Hap2	H2	HI2	STRiB	D2	G2	A1	T2	3
NN‐1	Hap1	H2	HI2	STRiA	D1	G1	A1	T1	2
NN‐2	Hap2	H2	HI2	STRiB	D2	G2	A1	T2	30
NNS‐1	Hap1	H2	HI2	STRiA	D1	G1	A1	T1	1
NNS‐2	Hap2	H2	HI2	STRiB	D2	G2	A1	T2	31
PZH‐1	Hap1	H2	HI2	STRiA	D1	G1	A1	T1	6
PZH‐2	Hap2	H2	HI2	STRiB	D2	G2	A1	T2	6
SM‐1	Hap1	H2	HI2	STRiA	D1	G1	A1	T1	4
SM‐2	Hap2	H2	HI2	STRiB	D2	G2	A1	T2	4
WZ‐1	Hap1	H2	HI2	STRiA	D1	G1	A1	T1	24
WZ‐2	Hap2	H2	HI2	STRiB	D2	G2	A1	T2	8
QZ‐1	Hap1	H1	HI1	STRiA	D1	G1	A1	T1	1
QZ‐2	Hap2	H2	HI2	STRiB	D2	G2	A1	T2	1
QZG‐1	Hap1	H1	HI1	STRiA	D1	G1	A1	T1	2
QZG‐2	Hap2	H2	HI2	STRiB	D2	G2	A1	T2	3
DY	Hap2	H2	HI2	STRiB	D2	G2	A1	T2	32
YL	Hap2	H2	HI2	STRiB	D2	G2	A1	T2	3
KM	Hap2	H2	HI2	STRiB	D2	G2	A1	T2	3
DYY	Hap2	H2	HI2	STRiB	D2	G2	A1	T2	32
LB	Hap2	H2	HI2	STRiB	D2	G2	A1	T2	16
QZH	Hap2	H2	HI2	STRiB	D2	G2	A1	T2	32
DZ	Hap2	H2	HI2	STRiB	D2	G2	A1	T2	4

### 3.4 Phylogenetic Analysis of Different Rickettsia Groups on the Basis of the 16S rRNA Sequences

**FIGURE 4:**
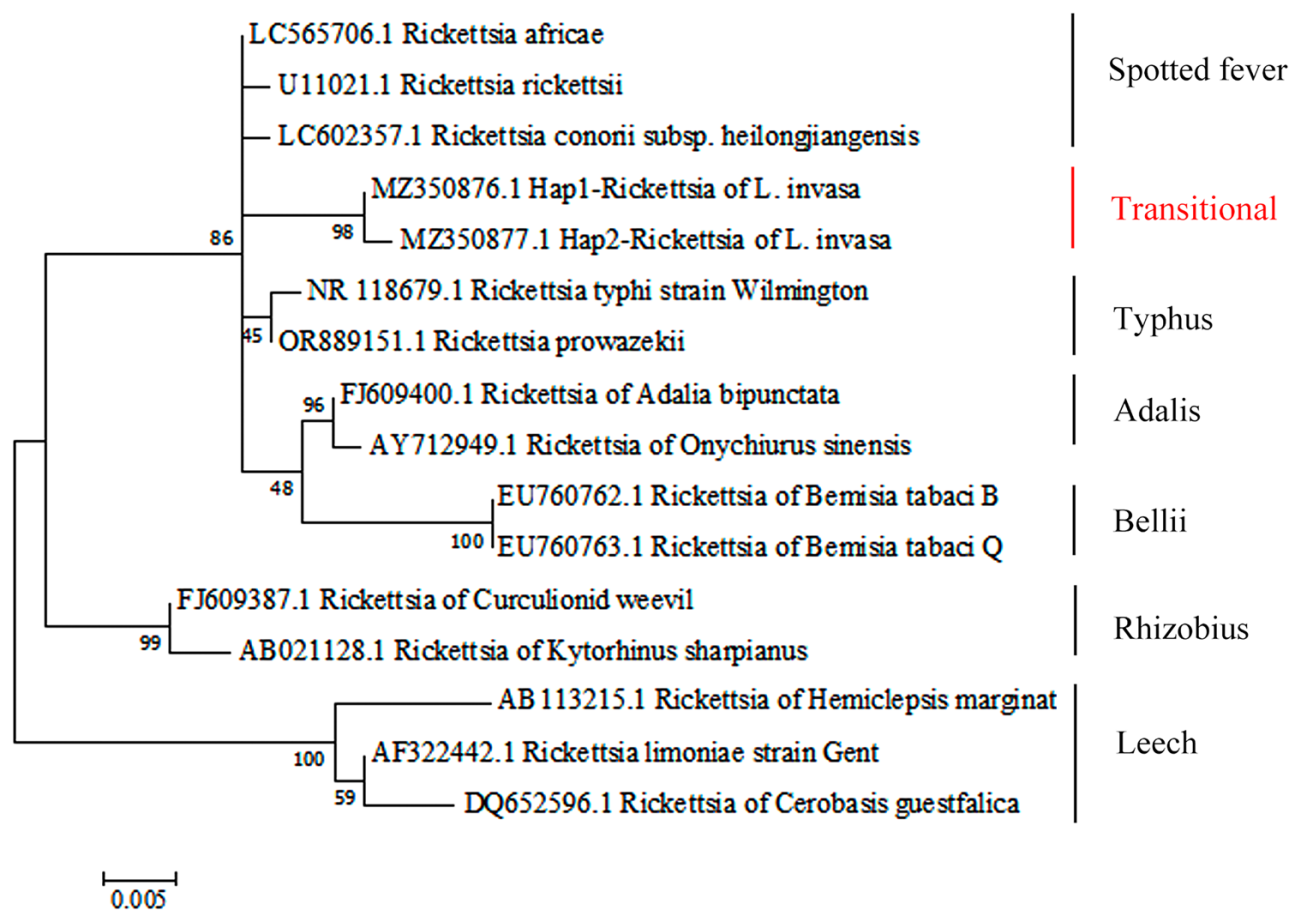
Phylogenetic analysis of different *Rickettsia* groups on the basis of the *16S* rRNA sequences. This phylogenetic tree was constructed on the basis of an aligned sequence of 484 amino acid residues. Values shown at branch nodes represent bootstrap support on the basis of 1000 replicates.

All 313 female *L. invasa* specimens examined were infected with *Rickettsia* (100% infection rate), whereas no infections were detected in males. Phylogenetic analysis of the *16S* rRNA gene revealed that the *Rickettsia* strain infecting *L. invasa* belongs to the Transitional group (Figure 4). It was classified between *Rickettsia felis* and *Rickettsia asembonensis*. The maximum Average Nucleotide Identity (ANI) with *R. asembonensis* was 95.1%, and the maximum digital DNA–DNA hybridization (dDDH) score with *R. felis* was 80.1%. The strain retained a degree of autonomous metabolic capability, suggesting it represents an evolutionary stage transitioning from a free‐living to an obligately parasitic lifestyle. Additionally, two haplotypes were identified for the *16S* rRNA, *gltA*, and *rpmE/tRNAf* ^*Met*^ genes, whereas only one haplotype was observed for the *atpA* gene. Multilocus sequence typing (MLST) of *Rickettsia* across 17 distinct *L. invasa* populations demonstrated co‐infection with two strains, designated STRiA and STRiB (Table 3). STRiA was detected in 10 populations (FCG, FCGS, GZ, NN, NNS, PZH, SM, WZ, QZ, and QZG), accounting for 23.0% of all infections, whereas STRiB occurred universally across all populations.

### 3.5 The Coevolution Between L. Invasa and Its Endosymbiont Rickettsia

The Hap2 haplotype was detected in all populations, yielding the *L. invasa COI* gene sequence. In contrast, the Hap1 haplotype was additionally identified in the FCG, FCGS, GZ, NN, NNS, PZH, SM, WZ, QZ, and QZG populations. Analysis of the obtained 556‐bp *28S* gene sequences revealed two distinct haplotypes. Haplotype H2 was present in all Chinese populations examined, whereas populations QZ, GZ, and QZG also exhibited haplotype H1. Similarly, two haplotypes of the *ITS* gene were observed among the *L. invasa* populations. Haplotype HI2 was detected universally across all populations, whereas haplotype HI1 was found exclusively in the QZ, GZ, and QZG populations (Table 3).

**FIGURE 5:**
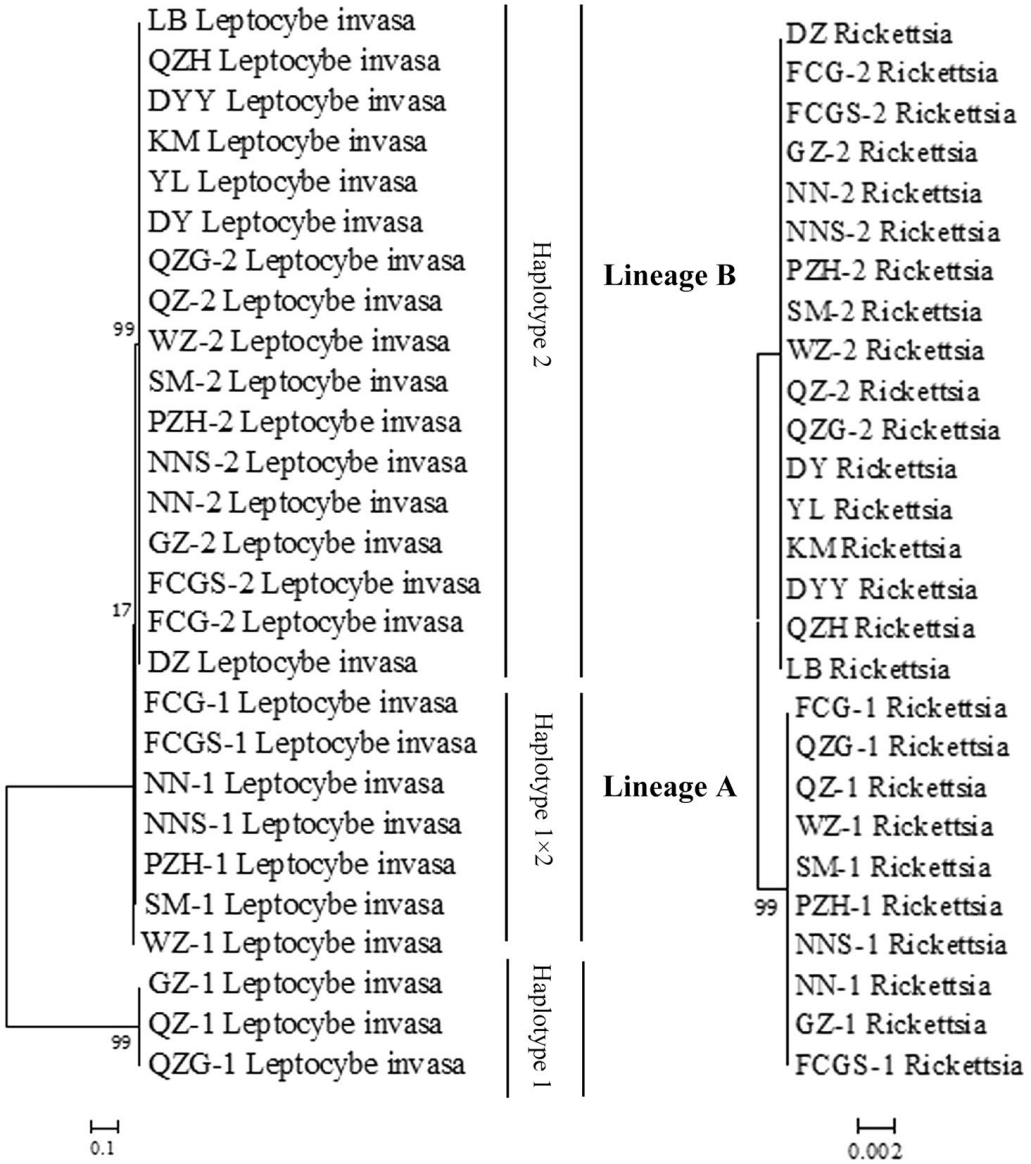
Phylogenetic patterns among *L. invasa* and corresponding *Rickettsia* strains. Left: Phylogenetic relationships among investigated *L. invasa* on the basis of *ITS*, *28S*, and *COI* sequences, the length of the trimmed amino acid sequence is 1615 bp. Right: Phylogeny of *Rickettsia* strains from *L. invasa*, with a trimmed amino acid sequence length of 2351 bp.

**FIGURE 6:**
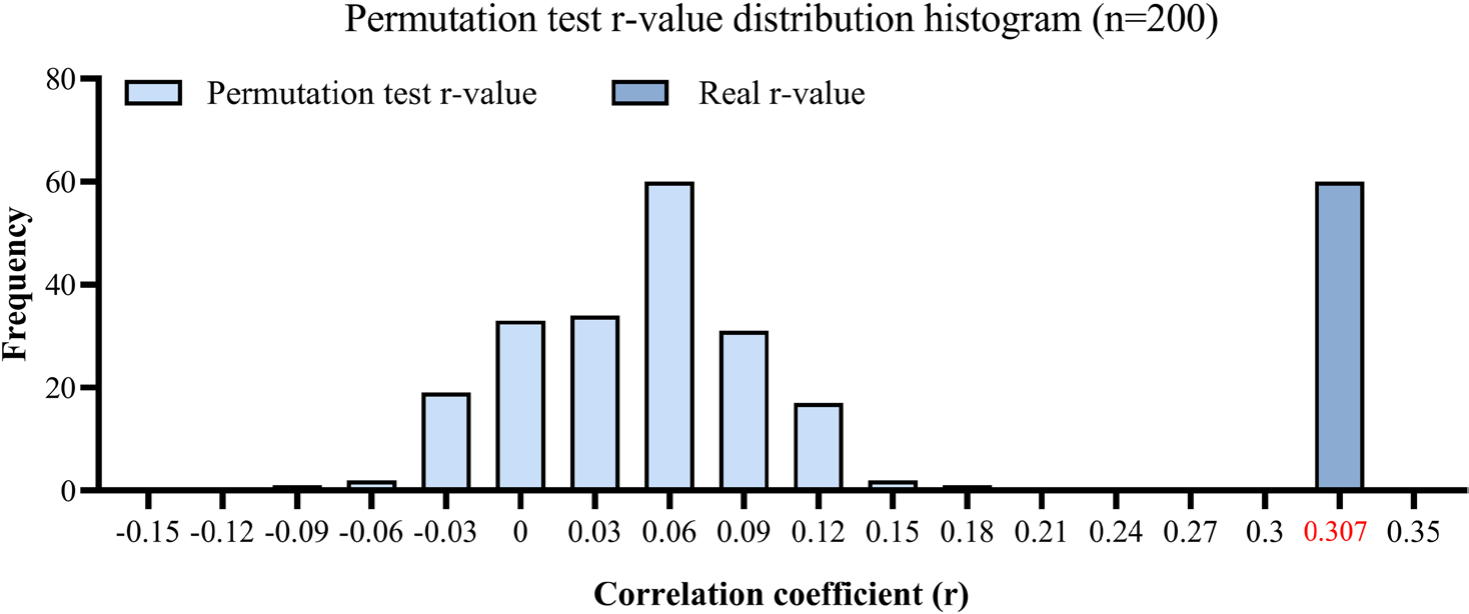
Frequency distribution of correlation coefficients (r) from the Mantel test permutation analysis. The histogram represents the null distribution generated from 200 permutations. Key parameters are as follows: Permutation‐derived *r*‐values ranged from −0.12 to 0.15 (mean = 0.0005). The observed correlation coefficient was *r* = 0.307 (*p* < 0.005).

Phylogenetic analysis of *COI*, *ITS*, and *28S* genes classified *L. invasa* specimens into three haplotypes (H1, H1 × 2, H2), which clustered into two distinct lineages. Only four *L*. *invasa* female specimens, assigned to haplotype H1, were detected in the QZ, QZG, and GZ populations. Seventy‐one specimens, corresponding to haplotype H1 × 2, were identified in the FCG, FCGS, NN, NNS, SM, WZ, and PZH populations. Notably, mito‐nuclear discordance was observed in the H1 × 2 lineage, with *COI* sequences aligning with cryptic lineage A, whereas *ITS* and *28S* sequences corresponded to cryptic lineage B. Additionally, the H2 lineage predominated among sampled populations in China, comprising 76% of all specimens. Consistency between *Rickettsia* and *L. invasa* was assessed through MLST of the *L. invasa COI* gene and *Rickettsia* loci, revealing congruent genetic patterns. Specifically, *Rickettsia* strains STRiA and STRiB exhibited host lineage specificity (Table 3). Concatenated phylogenetic analyses were performed using *L. invasa* sequences (*COI*, *ITS*, *28S*) and *Rickettsia* sequences (*16S* rRNA, *gltA*, *atpA*, *rpmE/tRNAf* ^*Met*^). Evaluation of the resultant evolutionary trees demonstrated congruent topologies between *L. invasa* and *Rickettsia*, indicative of coevolution (Figure 5). Following 200 permutations, a histogram of the resulting r‐values was constructed. The distribution of these permuted values was normal and did not exceed the observed correlation coefficient (Figure 6). Consequently, the Mantel test revealed a significant positive correlation between the genetic distance matrices of *L. invasa* and its endosymbiotic *Rickettsia* (*r* = 0.307, *p* < 0.005). This result provides strong statistical support for their coevolutionary relationship.

### 3.6 Effects of Antibiotic Treatment on the Longevity of L. Invasa and the Elimination of Rickettsia

#### 3.6.1 Effects of Antibiotic Treatment on the Longevity of *L. invasa*

**FIGURE 7:**
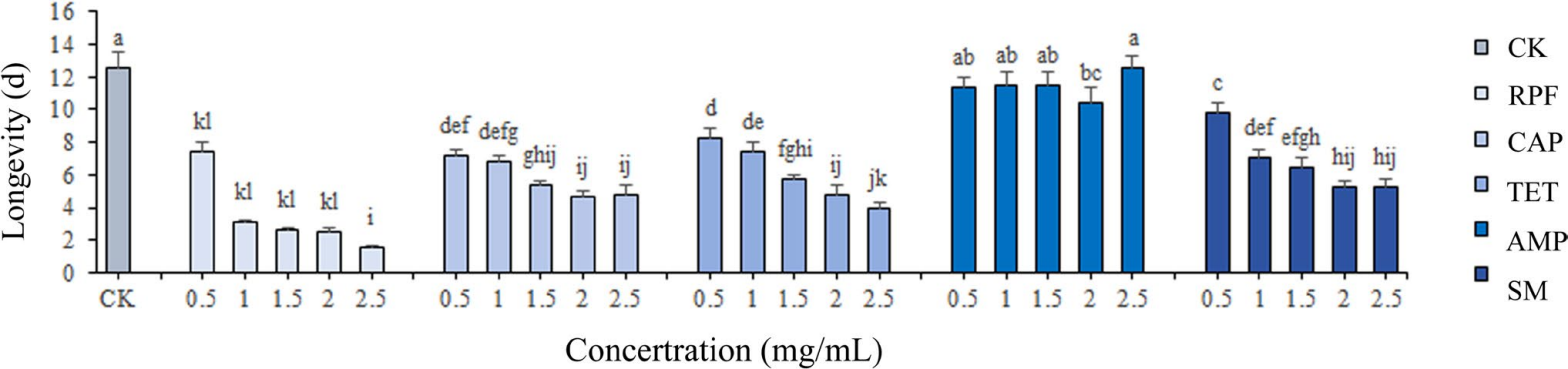
The influence of five antibiotics on female *L. invasa* longevity. The vertical bar in the figure represents the mean ± standard error (SE). Different letters mean significant difference from each other at *p* < 0.05 by LSD test (*n* = 30, *F* = 33.86).

Female *L. invasa* (lineage B) longevity under varying antibiotic treatments is presented in Figure 7, longevity decreased with increasing antibiotic concentrations. Ampicillin administration resulted in minimal longevity changes relative to CK, with 2.5 mg/mL AMP‐treated females exhibiting 12.56 d longevity (non‐significant difference from CK; *p* > 0.05), indicating negligible toxicity. Conversely, other antibiotics significantly reduced longevity, with rifampicin demonstrating the most pronounced effects: 1 mg/mL reduced longevity to 3.06 ± 0.23 d, whereas concentrations ≥ 1.5 mg/mL resulted in lifespans < 3 d, confirming strong toxicity. Tetracycline hydrochloride and streptomycin sulfate exhibited relatively lower toxicity at 0.5–1 mg/mL concentrations, maintaining longevity > 7 d.

#### 3.6.2 Effect of Antibiotic Treatment on the Removal *of Rickettsia* in *L. invasa*

**FIGURE 8:**
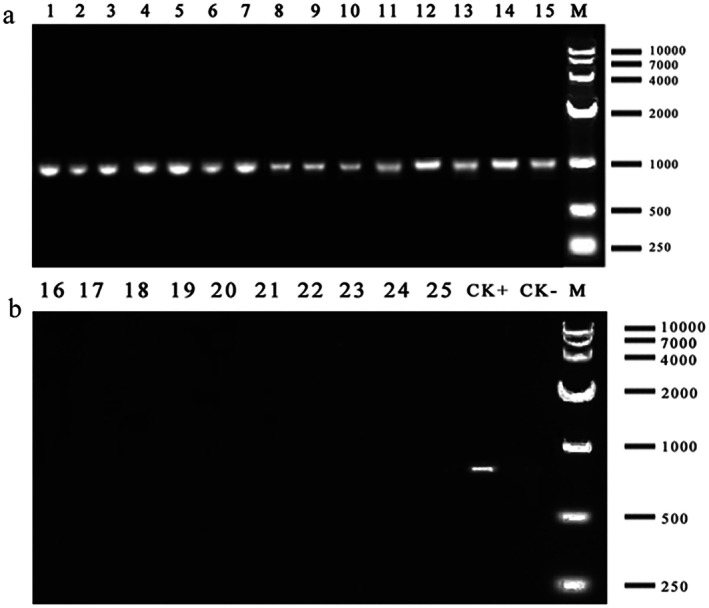
Effects of five antibiotics on removing *Rickettsia* from *L. invasa*. No. 1–5: 0.5–2.5 mg/mL SM; No. 6–10: 0.5–2.5 mg/mL CAP; No. 11–15: 0.5–2.5 mg/mL AMP; No. 16–20: 0.5–2.5 mg/mL RPF; No. 21–25: 0.5–2.5 mg/mL TET; M: Marker; CK+: Positive control; CK−: Negative Control (H_2_O).

*Rickettsia* infection status in *L. invasa* was assessed via PCR following 24‐h antibiotic exposure at concentrations of 0.5, 1, 1.5, 2, and 2.5 mg/mL, with an antibiotic‐free control. Figure 8 demonstrates that chloramphenicol, streptomycin sulfate, and ampicillin failed to eliminate *Rickettsia*. In contrast, rifampicin and tetracycline hydrochloride treatments yielded faint or absent *Rickettsia* amplification bands, indicating effective symbiont elimination (Figure 8). When integrated with longevity data (Figure 7), tetracycline hydrochloride at low concentrations (0.5–1 mg/mL) was determined to effectively clear *Rickettsia* while exhibiting relatively low host toxicity (*p* > 0.05).

### 3.7 Effects of Tetracycline Treatment on L. Invasa

**FIGURE 9:**
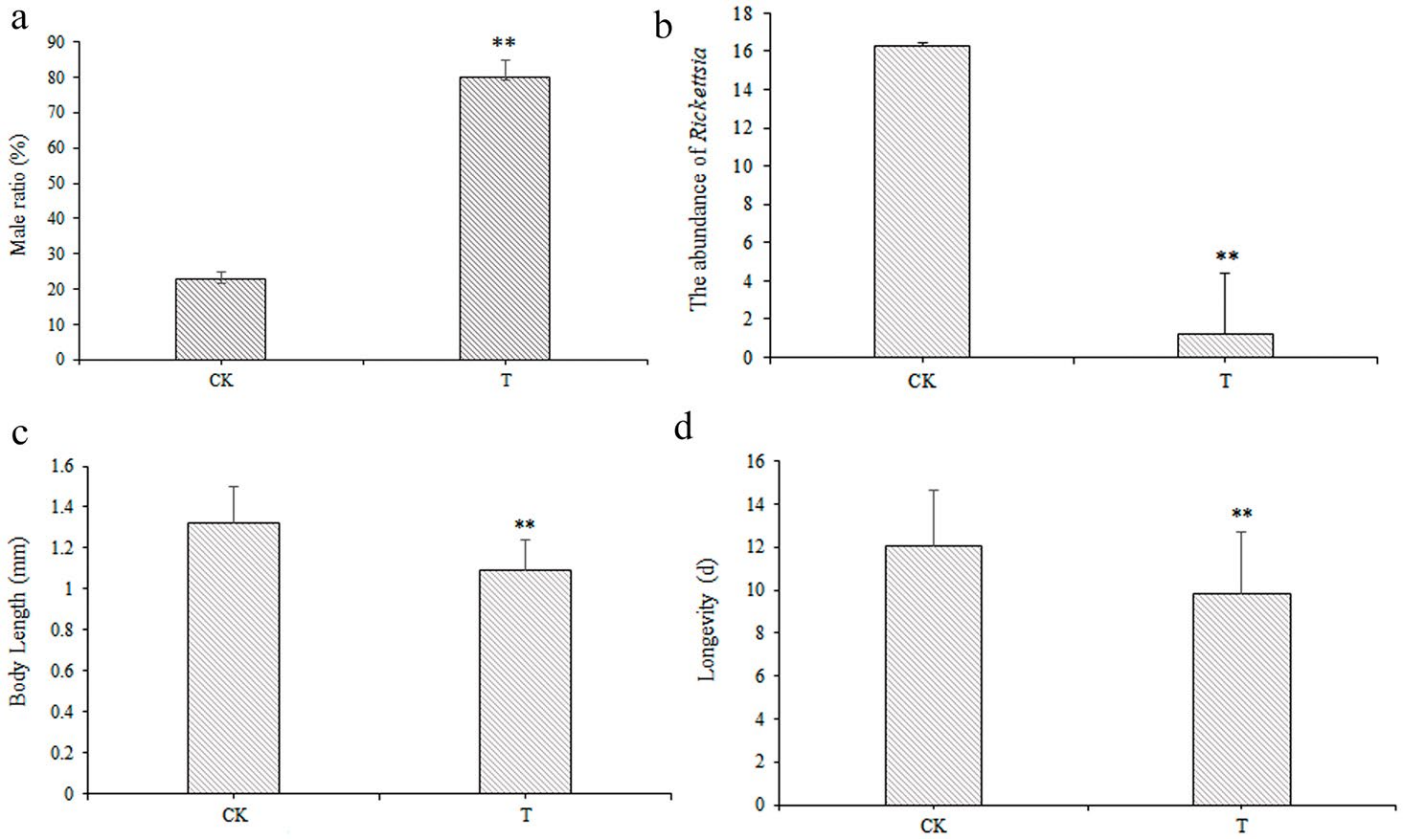
Effects of tetracycline treatment on physiological parameters of *L. invasa*. In the figure, CK denotes *L. invasa* without tetracycline treatment, whereas T denotes *L. invasa* following tetracycline treatment. (a) Male ratio of offspring after arcsine square‐root transformation (*n* = 30; Welch's *t* (41.33) = −10.996, *p* < 0.01). (b) Relative abundance of *Rickettsia* in females (*n* = 30; *t* (29.2) = −3.99, *p* < 0.01). (c) Female body length (*n* = 30; *t* (58) = 5.38, *p* < 0.01). (d) Longevity of female *L. invasa* (*n* = 30; *t* (58) = 3.10, *p* < 0.01). All datasets met assumptions of normality and homogeneity of variance unless otherwise noted; sex‐ratio data were transformed prior to analysis. Error bars represent Mean ± SE. Asterisks (**) indicate significant differences at *p* < 0.01 on the basis of independent‐samples *t*‐test.

Following treatment with 1.0 mg/mL tetracycline, a significant increase in male emergence was observed among newly emerged *L. invasa* (lineage B) offspring. As shown in Figure 9a, the male ratio in the tetracycline‐treated group attained 80.15% ± 4.72%, compared to 22.75% ± 2.23% in the untreated control group, representing a statistically significant divergence (*p* < 0.01). This shift indicates that the removal of *Rickettsia* via tetracycline treatment alters the sex ratio of *L. invasa* progeny. Quantitative PCR analysis confirmed that *Rickettsia* abundance in treated females was reduced to less than 1/15 of that in control insects, demonstrating a substantial yet incomplete elimination of the symbiont (Figure 9b). Morphometric measurements further revealed that the body length of female offspring from the treated group (1.09 ± 0.15 mm) was significantly shorter than that of controls (Figure 9c). Similarly, the longevity of tetracycline‐treated females (9.84 ± 2.86 days) was markedly reduced relative to control females (12.03 ± 2.61 days), with the difference being highly significant (*p* < 0.01; Figure 9d).

### 3.8 Comparative Transcriptomics

#### 3.8.1 Gene Function Annotation

This study employed antibiotic treatment to eliminate *Rickettsia* from *L. invasa* (lineage B), followed by transcriptome sequencing of symbiont‐carrying specimens. Sequencing yielded 37,854,780 to 47,814,634 high‐quality reads per sample, with average read lengths ranging from 148.72 to 149.27 bp. Quality metrics indicated Q20 values of 96.21%–96.98%, Q30 values of 90.33%–91.86%, GC content approximately 45%, and effective rates all exceeding 90% (Appendix S4), confirming data suitability for downstream analyses.

**FIGURE 10:**
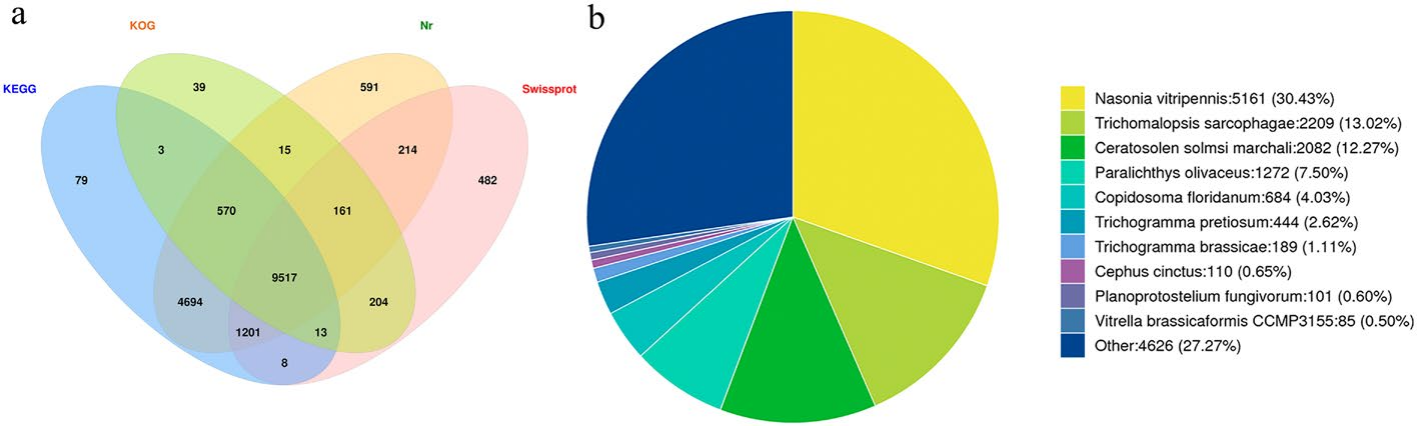
Gene function annotation. (a) Venn diagram of annotated unigenes in *L. invasa* transcriptome; (b) The species distribution on the gene sequence alignment of *L. invasa*.

Among 42,801 assembled unigenes, 17,791 (41.57%) were successfully annotated in public databases. The Nr database contained the most annotations (16,962 unigenes; 39.63%), followed by KEGG (16,085; 37.58%), Swiss‐Prot (11,800; 27.57%), and KOG (10,522; 24.58%). A total of 9517 unigenes received simultaneous annotation across all four databases (Figure 10a).

Nr database comparisons revealed highest sequence similarity with *Nasonia vitripennis* (5161 genes; 30.43%), followed by *Trichomatopsis sarcophagae* (2209; 13.02%), *Ceratosolen solmsi* (2082; 12.27%), *Paralichthys olivaceus* (1274; 7.50%), and *Copidosoma floridanum* (1274; 7.50%). Lower similarity was observed with *T. pretiosum* (444; 2.62%), *T. brassicae* (189; 1.11%), and *Cephus cinctus* (110; 0.65%) (Figure 10b).

#### 3.8.2 Differential Gene Analysis

**FIGURE 11:**
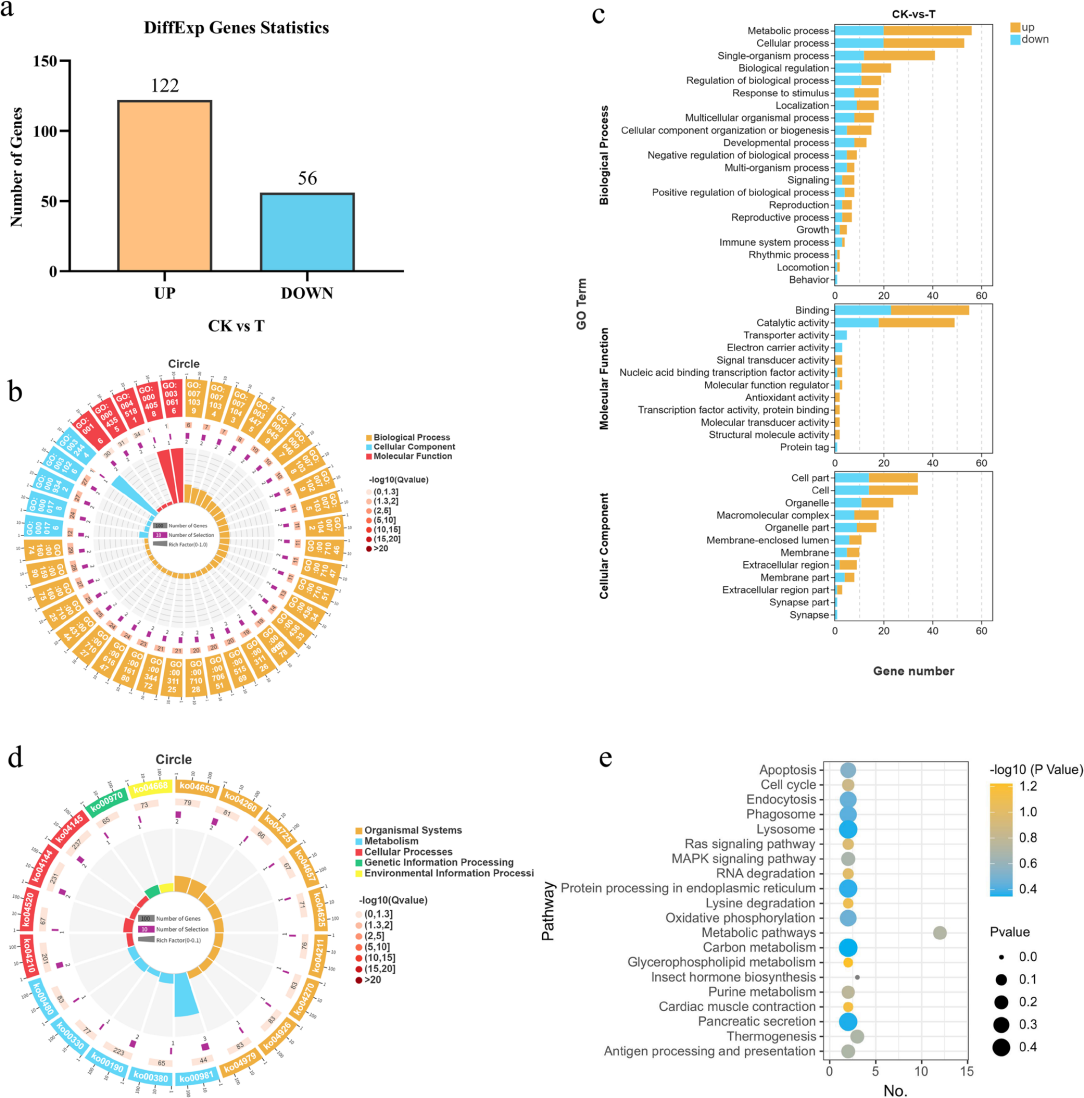
Differential gene enrichment analysis. (a) Statistical distribution of differentially expressed genes; (b) circular visualization of GO enrichment across three major categories; (c) histogram representation of GO secondary classification for differentially expressed genes; (d) circular visualization of KEGG metabolic pathway enrichment across five major categories; (e) bubble chart representation of KEGG secondary classification for differentially expressed genes.

The expression levels of homologous genes were compared between transcriptome data derived from *L. invasa* subjected to antibiotic‐mediated *Rickettsia* elimination and untreated, infected *L. invasa*, and DEGs were subsequently identified. Gene expression was quantified across samples using the FPKM metric. On the basis of the differential expression analysis, genes exhibiting a false discovery rate (FDR) value of less than 0.05 and an absolute log_2_ fold change (|log_2_FC|) greater than 1 were classified as differentially expressed. In comparison to the control, antibiotic treatment resulted in 178 significantly differentially expressed genes in *L. invasa*, comprising 122 up‐regulated and 56 down‐regulated genes (Figure 11a).

##### 3.8.2.1 GO Enrichment Analysis of Differential Genes

GO enrichment analysis of DEGs revealed their classification into three major ontological categories: biological processes, cellular components, and molecular functions (Figure 11b). These categories were further subdivided into 45 secondary classifications, distributed as 21, 12, and 12 subcategories, respectively. Within biological processes, metabolic processes contained the highest number of genes (56 total: 36 up‐regulated, 20 down‐regulated), followed by cellular processes (53 genes: 33 up‐regulated, 20 down‐regulated). Among cellular components, both cell and cell part classifications contained 34 genes each (20 up‐regulated, 10 down‐regulated). For molecular functions, binding activities represented the most populated category (55 genes: 32 up‐regulated, 23 down‐regulated), followed by catalytic activity (49 genes: 31 up‐regulated, 18 down‐regulated) (Figure 11c).

##### 3.8.2.2 KEGG Enrichment Analysis of Differential Genes

KEGG enrichment analysis of DEGs was conducted to identify metabolic pathway alterations in the *L. invasa* following *Rickettsia* elimination. DEGs were annotated within five primary KEGG pathway categories: Metabolism, Genetic Information Processing, Environmental Information Processing, Cellular Processes, and Organismal Systems, encompassing 24 subcategories and 96 specific metabolic pathways (Figure 11d). Annotation results revealed 42 differentially expressed genes enriched in Metabolism (25 pathways), 17 in Cellular Processes (11 pathways), 17 in Environmental Information Processing (15 pathways), 10 in Genetic Information Processing (8 pathways), and 41 in Organismal Systems (37 pathways). Comparative analysis between *Rickettsia*‐eliminated and control populations showed the metabolic pathway category contained the highest number of DEGs (8 up‐regulated, 4 down‐regulated), followed by insect hormone biosynthesis (3 genes) (Figure 11e). Additionally, these genes were enriched in multiple signaling pathways—including Ras, MAPK, NF‐κB, TGF‐β, TNF, Apelin, Sphingolipid, FoxO, Wnt, HIF‐1, Hippo, Hedgehog, cAMP, and PI3K‐Akt—and 13 immune system pathways, 12 of which were enriched for down‐regulated genes.

#### 3.8.3 Validation of RNA‐Seq Data by qRT‐PCR

**FIGURE 12:**
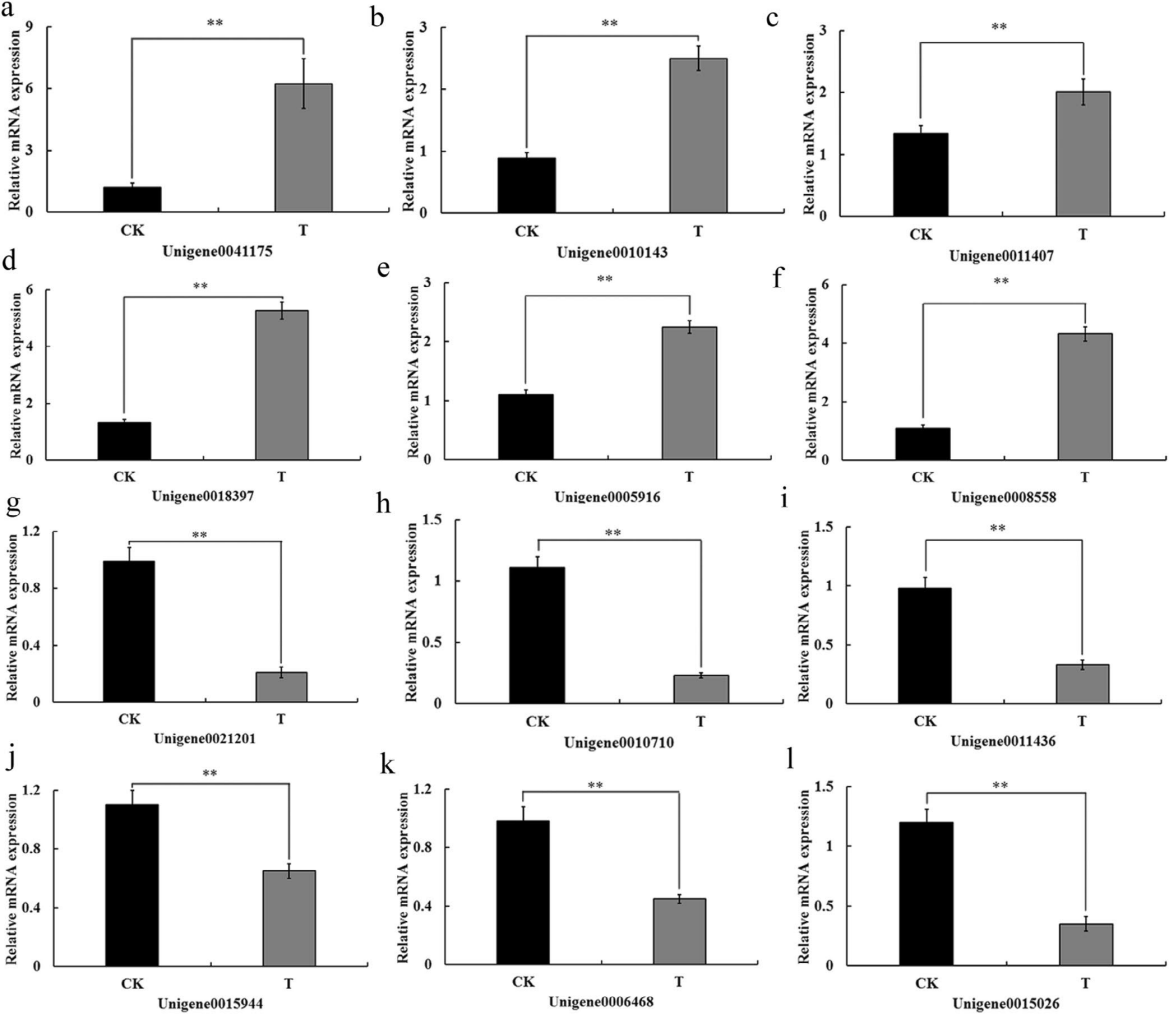
Validation of differentially expressed genes by qRT‐PCR. T: Female *L. invasa* by TET treatment; CK: Control Check. Panels (a–f): Up‐regulated genes; (g–l): Down‐regulated genes. Errorbars represent Mean ± SE. “**” represent the significant difference of male ratio at 0.01 level by *t* test. The validated genes, while randomly selected on the basis of expression variance, encompass a variety of functional categories relevant to stress response.

To validate the overall reliability and technical reproducibility of our transcriptomic sequencing data, we performed qRT‐PCR on 12 DEGs selected to represent a range of expression fold‐changes. As shown in Figure 12, the expression trends of these genes were highly consistent with the RNA‐seq results. It is noteworthy that these validated genes are involved in diverse biological processes, including chitin metabolism, signal transduction, and cellular oxidative respiration, indicating that our transcriptome data faithfully captured broad transcriptional responses to TET treatment.

## 4 Discussion

### 4.1 Dynamic Changes of Rickettsia Abundance in L. Invasa

The abundance of symbiotic bacteria in insects is often dependent on the developmental stage of the host. Previous studies have demonstrated that *Wolbachia* is present in both the egg and adult stages of *Episyrphus balteatus*, with the highest relative abundance observed during the egg stage. In contrast, the symbiotic bacterium *Rickettsia* in *Bemisia tabaci* exhibits a dynamic developmental distribution, spreading across embryonic and pupal stages and ultimately accumulating predominantly in the abdominal midgut during adulthood (Gottlieb et al. 2006; Wang et al. 2024). In this study, it was found that *Rickettsia* abundance in female adults of *L. invasa* was significantly higher than in larvae and pupae from 1 to 6 days post‐emergence; furthermore, abundance in young larvae was significantly greater than in mature larvae (Figure 2a). This pattern supports the hypothesis of vertical *Rickettsia* transmission through eggs in *L. invasa*. Tissue distribution analysis further revealed that *Rickettsia* abundance in the abdomen was significantly higher than in the head and thorax (Figure 2b). This distribution pattern is consistent with the enrichment characteristics of various endosymbionts known to regulate host reproduction, such as *Wolbachia* in *T. confusum* (Shen et al. 2016). As a reproductive manipulator, the high abundance of *Rickettsia* in the abdomen is likely related to its localization within the host's ovarian tissue. This localization facilitates its vertical transmission and persistence within the host population by inducing a reproductive advantage. Collectively, these findings provide a new experimental basis for understanding the mechanisms of persistent infection and transmission of *Rickettsia* in *L. invasa* through an analysis of its spatiotemporal dynamics and tissue distribution.

### 4.2 Two Lineages L. Invasa of Discovered in China and Lineage A Was First Reported

Previous studies have documented the genetic diversity of *L. invasa*. On the basis of *mtCOI* sequence analysis, *L. invasa* has been divided into two distinct lineages. Specifically, lineage A dominates populations in Africa, the Middle East, and the Mediterranean region, whereas lineage B is prevalent and frequently collected in Southeast Asia and Australia (Dittrich‐Schröder et al. 2018; Nugnes et al. 2015). This study revealed the coexistence of both lineages within China, with lineage A being reported here for the first time. Moreover, lineage B was detected in all sampled populations across China, indicating its dominance in the region (Figure 3). Indeed, lineage B has been the sole lineage reported and widely collected in China since the wasp's initial detection in 2007. However, a significant number of lineage A individuals were also detected in this study, a finding potentially influenced by the extensive sampling range. Seventeen distinct populations were sampled, encompassing nearly all known regions infested by *L. invasa* in southwestern, southeastern, and southern China. This comprehensive sampling provides a more accurate representation of the lineage distribution of this invasive wasp within the country. Furthermore, this refined assessment of *L. invasa*'s cryptic distribution within China also corroborates the widespread global invasion of lineage A.

Hybridization between lineages may enhance adaptability through hybrid vigor, particularly during initial invasion stages, conferring advantages to invasive species (Rabosky et al. 2009; Verhoeven et al. 2011). The detection of two distinct lineages of *L. invasa* in China necessitates further investigation into the relationship between this hybrid introgression and invasion dynamics. Additionally, two parasitoid biological control agents, *Quadrastichus mendeli* Kim & La Salle and *Selitrichodes kryceri* Kim & La Salle (Hymenoptera: Eulophidae), have demonstrated efficacy in reducing damage caused by *L. invasa* lineage A (Salle 2008). However, the efficacy of these biological control agents against *L. invasa* lineage B remains undetermined. In a previous study, the parasitoid *Megastigmus sichuanensis* was identified in Sichuan Province, China; while it exhibits potential for suppressing *L. invasa* infestations, its efficacy requires further investigation (Zheng et al. 2016).

### 4.3 The Phenomenon of Mitochondrial‐Nuclear Discordance Exists in L. Invasa

Three distinct haplotypes of *L. invasa* were identified in China on the basis of analyses of the *COI*, *ITS*, and *28S* genes (Figure 5). Lineage A, reported here for the first time, exhibits mitochondrial‐nuclear discordance (Table 3). Mitochondrial‐nuclear discordance has also been documented in *Frankliniella occidentalis* populations in China (Yang et al. 2012). This discordance may arise from differential evolutionary rates and genetic patterns between genomes (Kawasaki et al. 2016; Shaikevich et al. 2019; Sontowski et al. 2015; Spottiswoode et al. 2011). Maternally inherited mitochondrial genomes are more susceptible to random mutations, natural selection, genetic drift, and other evolutionary forces, resulting in greater variation across geographical populations and species compared to nuclear genomes. Host plant diversity may also contribute to this phenomenon (Li et al. 2014; Nault et al. 2006). For example, *Thrips tabaci* lineage T is associated with tobacco, whereas lineages L1 and L2 are associated with chives (Toda and Murai 2007). In this study, *L. invasa* specimens were collected from five host plants (Table 1). Two lineages were detected on four hosts ( *Eucalyptus globulus* yielded only four wasps, all belonging to lineage B). Consequently, no correlation between host plant identity and mitochondrial‐nuclear discordance was observed in this study.

An alternative and potentially more probable explanation involves hybridization and introgression between the two *L. invasa* lineages, resulting in mitochondrial‐nuclear discordance. Reproductive strategy is widely recognized as a principal factor influencing genetic differentiation in insect pests (Kobayashi et al. 2013; Li et al. 2020). This perspective is supported by Peng et al., whose SSR analysis documented mitochondrial‐nuclear discordance and indicated bidirectional introgression between *L. invasa* lineages (Peng et al. 2021). Although parthenogenesis represents the primary reproductive mode in *L. invasa*, males have been documented in several regions, particularly in China (Guo et al. 2020; Huang et al. 2019; Nugnes et al. 2015). Consequently, sexual reproduction constitutes a significant secondary reproductive strategy (Zheng, Yang, et al. 2014). Both reproductive strategies can enhance genetic diversity and population size in *L. invasa*, thereby facilitating adaptation to diverse environmental conditions. Relative to populations in the Middle East and Mediterranean regions, Southeast Asian populations exhibit a higher frequency of *L. invasa* males. In the present study, males were similarly detected, with DNA barcoding revealing representation of both lineages among Chinese specimens (Figure 3). Thus, this study proposes that hybridization between lineages may contribute to mitochondrial‐nuclear discordance.

### 4.4 Phylogenetic Congruence Between Rickettsia and L. Invasa

In this study, a 100% *Rickettsia* infection rate was observed among females across all 17 sampled populations in China, indicating successful colonization of *L. invasa* by this endosymbiont (Table 2). Conversely, *Rickettsia* was undetectable in all male specimens examined. As a maternally inherited intracellular endosymbiont, *Rickettsia* influences host insect reproduction through mechanisms including parthenogenesis induction (PI) and male killing (MK) (Adachi et al. 2008). Previous research has demonstrated that *Rickettsia*‐infected *Pnigalio soemius* exclusively produced female offspring, while male progeny was obtained only after *Rickettsia* elimination from adult wasps (Giorgini et al. 2010). Nugnes et al. documented *Rickettsia* localization within female reproductive tissues; however, its functional role in *L. invasa* requires further investigation (Nugnes et al. 2015).

Previous studies have demonstrated that infection by inherited endosymbionts can reduce mtDNA variation and affect host genetic diversity, as documented in *Polytremis* (Lepidoptera: Hesperiidae) and *Plutella xylostella* L. (Lepidoptera: Plutellidae) (Jiang et al. 2018; Zhu et al. 2023). Endosymbionts can drive natural selection on mtDNA through their transmission dynamics and capacity for multiple infections, thereby influencing host evolution (Kawasaki et al. 2016; Sontowski et al. 2015; Yu et al. 2011). Furthermore, endosymbionts can induce selective sweeps that reduce host mitochondrial diversity (Ma et al. 2025). Additional evidence from endosymbiont‐host coevolution systems, including *Orseolia oryzae* (Behura et al. 2001) and *Brontispa longissima* (Ali et al. 2018), provides further support for this phenomenon. Variation among *Rickettsia* strains in their ability to manipulate host reproduction may consequently influence mtDNA diversity (Yu et al. 2011). The topological congruence between *L. invasa* and *Rickettsia* phylogenies implies a potential association between *Rickettsia* infection and mtDNA diversity in this host (Figure 5). Unfortunately, no *Rickettsia*‐uninfected females have been identified to date; consequently, the relationship between *Rickettsia* infection and the genetic diversity of *L. invasa* requires further investigation. Moreover, this association may indicate vertical rather than horizontal transmission of *Rickettsia* (Nugnes et al. 2015). Although horizontal transfer has been documented across different hosts, including *Bemisia tabaci* (Yadav et al. 2024) and the sweetpotato whitefly (Chiel et al. 2009), vertical cytoplasmic inheritance remains the dominant transmission strategy (Caspi‐Fluger et al. 2012; Wierz et al. 2024). Indeed, current evidence provides no indication of horizontal *Rickettsia* transmission in *L. invasa* (Gualtieri et al. 2017).

### 4.5 Functional Analysis of Rickettsia in L. Invasa on the Basis of Transcriptome

*L. invasa* represents a severely feminized invasive pest species, typically exhibiting sex ratios (male:female) ranging from 1:2.1 to 1:5.5, with extreme ratios reaching 1:23.2–1:195 in certain populations (Zheng et al. 2018). As *Rickettsia* is known to be enriched in female ovarian tissue and has been demonstrated to regulate parthenogenesis in various insect species through meiotic alteration, we investigated its role in reproductive manipulation (Figure 2b). Experimental removal of *Rickettsia* significantly increased male offspring proportion, confirming its critical role in reproductive regulation and suggesting its function in maintaining female‐biased populations through parthenogenesis induction (Figure 9a). Beyond reproductive manipulation, *Rickettsia* may enhance host environmental resilience and fitness during invasion events (Majerus and Majerus 2010). Previous research by Himler et al. (Himler et al. 2011) demonstrated that *Rickettsia* infection in B‐type tobacco whiteflies shortened developmental duration while significantly increasing oviposition rate and survival. Correspondingly, our study revealed that *Rickettsia* elimination significantly reduced both longevity and body size in *L. invasa* (Figure 9c,d). Notably, tetracycline's broad‐spectrum activity against chlamydia, spirochetes, and Gram‐positive/negative bacteria introduces potential confounding effects through elimination of non‐target microbiota. While antibiotic feeding remains the primary methodology for symbiont elimination, future studies should develop *Rickettsia*‐specific removal techniques to eliminate potential confounding effects of microbiome disruption on reproductive development.

Transcriptome sequencing revealed that DEGs were predominantly enriched in metabolic pathways, insect hormone biosynthesis, and multiple immune signaling pathways, indicating that *Rickettsia* significantly influences growth, development, and reproductive metabolism in *L. invasa*. Specifically, 13 annotated immune system pathways were identified, 12 of which—including both Toll‐like and NOD‐like receptor signaling pathways—exhibited down‐regulated expression patterns (Figure 11). This widespread immunosuppression likely accounts for the observed reduction in adult lifespan, increased proportion of males, and decreased body size following tetracycline‐mediated elimination of *Rickettsia*. *Rickettsia* is proposed to form an “immune shield” for the host, potentially enhancing host defense against pathogenic microorganisms and parasitic natural enemies, thereby increasing adaptive capacity and invasive spread potential. Accordingly, pest management strategies could explore targeted interference against *Rickettsia* to reduce the basal immune level of pest populations, thereby enhancing susceptibility to biological control agents, including pathogenic fungi, bacteria, and parasitoids. This finding is consistent with research on the related endosymbiont *Wolbachia*, which is known to influence host fitness through mechanisms including the regulation of female reproduction and the reduction of host lifespan. Transcriptomic analyses comparing infected and uninfected hosts reveal that differentially expressed genes are enriched in pathways related to detoxification, redox processes, metabolism, and immunity. These functional shifts underpin the consideration of such endosymbionts as potential agents for biological control (Gong et al. 2020). Moreover, the observed down‐regulation of key genes within major immune pathways (e.g., the Toll pathway) provides potential molecular targets for gene‐targeting approaches such as RNA interference, enabling direct disruption of pest immune defenses and improving biocontrol efficacy. For instance, the *PxRan* gene in the diamondback moth enhances tolerance to deltamethrin through activation of Toll‐related pathways (Zhang 2021). Zhang et al. identified 12 key Toll pathway genes in peach aphids that were significantly up‐regulated under microbial stress, and subsequent RNA interference experiments combined with pathogen exposure confirmed that silencing these genes severely compromises aphid antibacterial defenses (Zhang et al. 2025).

## 5 Conclusions

This study identified *Rickettsia* as the dominant endosymbiont in female *L. invasa*, primarily localized within abdominal tissues, suggesting a coevolutionary relationship with the host and supporting its maternal inheritance pattern. Antibiotic elimination of *Rickettsia* using tetracycline resulted in significantly increased male offspring production, indicating this endosymbiont plays a crucial role in offspring sex ratio determination. These findings suggest that *Rickettsia* infection represents a primary factor in the observed feminization of *L. invasa* populations and supports thelytokous parthenogenesis as its predominant reproductive mode. Furthermore, *Rickettsia* elimination correlated with reduced offspring longevity and diminished body size. Transcriptomic analysis revealed significant enrichment of differentially expressed genes in insect hormone biosynthesis and metabolic pathways, with functional annotations indicating roles in nutrition, energy metabolism, and immune defense mechanisms. Collectively, these results demonstrate that *Rickettsia* significantly influences growth, development, metabolic processes, and immunological functions in *L. invasa*. This endosymbiont consequently plays an integral role in host invasion, dispersal capacity, and environmental adaptation, warranting further investigation into its mechanisms governing reproductive strategy manipulation.

## Author Contributions

**Xiu Xu:** data curation (equal), project administration (equal), software (equal), supervision (equal), visualization (equal), writing – original draft (equal), writing – review and editing (equal). **Leming Zhou:** formal analysis (equal), project administration (equal), resources (equal), writing – review and editing (equal). **Jinting Xie:** software (equal), validation (equal). **Junjue Li:** software (equal), validation (equal). **Chunhui Guo:** conceptualization (equal), methodology (equal), supervision (equal). **Zhende Yang:** conceptualization (equal), funding acquisition (equal), investigation (equal).

## Funding

The research was financially supported by the National Natural Science Foundation of China (32360390; 31971664) and Guangxi Natural Science Foundation (2018GXNSFDA281004).

## Conflicts of Interest

The authors declare no conflicts of interest.

## Supporting information


**Appendices S1–S5:** ece373066‐sup‐0001‐AppendicesS1‐S5.docx.

**Data S1:** ece373066‐sup‐0002‐DataS1.rar.

**Data S2:** ece373066‐sup‐0003‐DataS2.rar.

## Data Availability

All the required data are uploaded as Supporting Information.
