# Supplementary material for: Coevolution and Functional Effects of Endosymbiotic Rickettsia in Leptocybe invasa Fisher & LaSalle (Hymenoptera: Eulophidae) Across China
Source: Ecol Evol. 2026 Feb 10;16(2):e73066. doi: 10.1002/ece3.73066 (PMC12891809; doi:10.1002/ece3.73066)
Supplement: Supplementary file 1 — Appendices S1–S5: ece373066‐sup‐0001‐AppendicesS1‐S5.docx. [file ECE3-16-e73066-s002.docx]

Appendix

**Appendix 1** Environmental data in different populations.

| Environmental factor | DY | FCG | FCGS | GZ | NN | NNS | PZH | QZ | SM | WZ |
| --- | --- | --- | --- | --- | --- | --- | --- | --- | --- | --- |
| Rainfall (mm) | 68.04 | 120.52 | 107.5 | 69.9 | 143.61 | 141.03 | 122.88 | 101.53 | 101.53 | 107.5 |
| Annual high temperature (°C) | 20.75 | 24.18 | 26.45 | 27.73 | 26.61 | 25.51 | 27.06 | 26.74 | 26.74 | 26.45 |
| Annual low temperature (°C) | 13.38 | 16.28 | 18.68 | 15.53 | 18.11 | 15.72 | 18.18 | 18.11 | 18.11 | 18.68 |

**Appendix 2** Primer sequence information used in paper.

| Gene | Primers | Primers Sequence |
| --- | --- | --- |
| *β-actin* | β-actin-F | GCCGTGTTCCCGTCCATCGT |
|  | β-actin-R | GCGGTTGCCATTTCCTGCTC |
| *qgltA* | qgltA-F | AGAATTACCAAGCATCGAGCAG |
|  | qgltA-R | GCCGCAAGCATAATAGCCATA |
| *COI* | LCO1490 | 5'-GGTCAACAAATCATAAAGATATTGG-3' |
|  | HCO2198 | 5'-TAAACTTCAGGGTGACCAAAAAATCA-3' |
| *28S* | D2F | 5'-AGTCGTGTTGCTTGATAGTGCAG-3' |
|  | D2R | 5'-TTGGTCCGTGTTTCAAGACGGG-3' |
| *ITS2* | *ITS*2F | 5'-TGTGAACTGCAGGACACA-3' |
|  | *ITS*2R | 5'-GTCTTGCCTGCTCTGAG-3' |
| *16S rRNA* | RbF | 5'-GCTCAGAACGAACGCTATC-3' |
|  | RbR | 5'-GAAGGAAAGCATCTCTGC-3' |
| *gltA* | CS113d | 5'-GTAAAGTATATTCTGAAGCG-3' |
|  | CS715R | 5'-CATTTTGCTCATGATCAGC-3' |
| *atpA* | *atpA*-F | 5'-AGTACAGACATATCGAGATGA-3' |
|  | *atpA*-R | 5'-CGACTTACCGAAATACCGAC-3' |
| *rpmE-tRNAf^Met^* | *rpmE*-F | 5'-TTCCGGAAATGTAGTAAATCAATC-3' |
|  | *rpmE*-R | 5'-TCAGGTTATGAGCCTGACGA-3' |

**Appendix 3** GenBank accession numbers of *Rickettsia* sequences and *L. invasa* populations.

| Population | *Leptocybe invasa* | | | *Rickettsia* | | | |
| --- | --- | --- | --- | --- | --- | --- | --- |
|  | *COI* | *28S* | *ITS* | *16S* | *gltA* | *atpA* | *rpmE* |
| FCG-1 | MZ311551 | MZ325352 | MZ343213 | MZ350876 | MZ364521 | MZ388520 | MZ343305 |
| FCG-2 | MZ311552 | MZ325353 | MZ343214 | MZ350877 | MZ364522 | MZ388521 | MZ343306 |
| FCGS-1 | MZ311553 | MZ325354 | MZ343215 | MZ350878 | MZ364523 | MZ388522 | MZ343307 |
| FCGS-2 | MZ311554 | MZ325355 | MZ343216 | MZ350879 | MZ364524 | MZ388523 | MZ343308 |
| GZ-1 | MZ311555 | MZ325356 | MZ343217 | MZ350880 | MZ364525 | MZ388524 | MZ343309 |
| GZ-2 | MZ311556 | MZ325357 | MZ343218 | MZ350881 | MZ364526 | MZ388525 | MZ343310 |
| NN-1 | MZ311557 | MZ325358 | MZ343219 | MZ350882 | MZ364527 | MZ388526 | MZ343311 |
| NN-2 | MZ311558 | MZ325359 | MZ343220 | MZ350883 | MZ364528 | MZ388527 | MZ343312 |
| NNS-1 | MZ311559 | MZ325360 | MZ343221 | MZ350884 | MZ364529 | MZ388528 | MZ343313 |
| NNS-2 | MZ311560 | MZ325361 | MZ343222 | MZ350885 | MZ364530 | MZ388529 | MZ343314 |
| PZH-1 | MZ311561 | MZ325362 | MZ343223 | MZ350886 | MZ364531 | MZ388530 | MZ343315 |
| PZH-2 | MZ311562 | MZ325363 | MZ343224 | MZ350887 | MZ364532 | MZ388531 | MZ343316 |
| SM-1 | MZ311565 | MZ325366 | MZ343227 | MZ350890 | MZ364535 | MZ388534 | MZ343319 |
| SM-2 | MZ311566 | MZ325367 | MZ343228 | MZ350891 | MZ364536 | MZ388535 | MZ343320 |
| WZ-1 | MZ311567 | MZ325368 | MZ343229 | MZ350892 | MZ364537 | MZ388536 | MZ343321 |
| WZ-2 | MZ311568 | MZ325369 | MZ343230 | MZ350893 | MZ364538 | MZ388537 | MZ343322 |
| QZ-1 | MZ311563 | MZ325364 | MZ343225 | MZ350888 | MZ364533 | MZ388532 | MZ343317 |
| QZ-2 | MZ311564 | MZ325365 | MZ343226 | MZ350889 | MZ364534 | MZ388533 | MZ343318 |
| QZG-1 | MZ311569 | MZ325370 | MZ343231 | MZ350894 | MZ364539 | MZ388538 | MZ343323 |
| QZG-2 | MZ311570 | MZ325371 | MZ343232 | MZ350895 | MZ364540 | MZ388539 | MZ343324 |
| DY | MZ311550 | MZ325351 | MZ343212 | MZ350875 | MZ364520 | MZ388519 | MZ343304 |
| YL | MZ311571 | MZ325372 | MZ343233 | MZ350896 | MZ364541 | MZ388540 | MZ343325 |
| KM | MZ311572 | MZ325373 | MZ343234 | MZ350897 | MZ364542 | MZ388541 | MZ343326 |
| DYY | MZ311573 | MZ325374 | MZ343235 | MZ350898 | MZ364543 | MZ388542 | MZ343327 |
| LB | MZ311575 | MZ325376 | MZ343237 | MZ350900 | MZ364545 | MZ388544 | MZ343329 |
| QZH | MZ311576 | MZ325377 | MZ343238 | MZ350901 | MZ364546 | MZ388545 | MZ343330 |
| DZ | MZ311574 | MZ325375 | MZ343236 | MZ350899 | MZ364544 | MZ388543 | MZ343328 |

**Appendix 4** Basic information of transcriptome sequencing in the *L. invasa*.

| Sample | Raw Tags | Effective Tags | AvgLen (bp) | GC (%) | Q20 (%) | Q30 (%) | Effective (%) |
| --- | --- | --- | --- | --- | --- | --- | --- |
| CK1 | 42159586 | 42032022 | 149.13 | 43.49 | 96.98 | 91.86 | 91.68 |
| CK2 | 37981652 | 37854780 | 149.26 | 43.79 | 96.56 | 90.99 | 90.81 |
| CK3 | 40609768 | 40190596 | 148.72 | 47.36 | 96.72 | 91.36 | 90.98 |
| T1 | 48166876 | 47814634 | 148.74 | 47.37 | 96.48 | 90.91 | 90.58 |
| T2 | 43322234 | 43171626 | 149.08 | 43.16 | 96.21 | 90.33 | 90.14 |
| T3 | 45139372 | 45005050 | 149.27 | 43.32 | 96.67 | 91.19 | 91.02 |

**Appendix 5** Primer sequences used for qRT-PCR validation.

| Gene | Primers Sequence |
| --- | --- |
| Unigene0021201 | F: 5'-CCAATCAAAGCGCAGTCT-3' |
|  | R: 5'-ATGTCGGCAGCGAGTATG-3' |
| Unigene0041175 | F: 5'-CAGTTTCTTGGGCTATTC-3' |
|  | R: 5'-CTACATACATTTGTGGGTTT-3' |
| Unigene0010143 | F: 5'-TGGTTCGACCTTCCCTCC-3' |
|  | R: 5'-TTCTCCACCGCCTTGTTT-3' |
| Unigene0005916 | F: 5'-ATCACAAACGTGCATCTA-3' |
|  | R: 5'-TGTAGCCAGAATCCAGTA-3' |
| Unigene0010710 | F: 5'-ACTTCGGCAGTCAGGTAT-3' |
|  | R: 5'-TTCAGTTCCCAGTTGGTC-3' |
| Unigene0011436 | F: 5'-CGCAATCAATAAATCCTG-3' |
|  | R: 5'-GCTCTGTACGAAACGCTA-3' |
| Unigene0015026 | F: 5'-TCCTTCGTTTTCATAGTG-3' |
|  | R: 5'-ATAAGAGCAAGCATCAGT-3' |
| Unigene0006468 | F: 5'-CAACATTCATCCTTCTCA-3' |
|  | R: 5'-AGCTATTTCAAATCCCAA-3' |
| Unigene0015944 | F: 5'-AATGATCGGGAGTTATTCA-3' |
|  | R: 5'-ATTTGTCTGCCAGTAGCG-3' |
| Unigene0015849 | F: 5'-ATGAAGAGCGTGATTGCC-3' |
|  | R: 5'-GATCCCTTGCCACCGTAA-3' |
| Unigene0018397 | F: 5'-CGTCGGTGCTTAAATCTG3' |
|  | R: 5'-TGAACCTTCCTTGGCGTA-3' |
| Unigene0008558 | F: 5'-AGGATGATTCGGTGCTAA-3' |
|  | R: 5'-GTTCCTCGGAGTCGTTCA-3' |
| β-Actin | F: 5'-GCCGTGTTCCCGTCCATCGT-3' |
|  | R: 5'-GCGGTTGCCATTTCCTGCTC-3' |
